# Hypomethylation of a LINE-1 Promoter Activates an Alternate Transcript of the MET Oncogene in Bladders with Cancer

**DOI:** 10.1371/journal.pgen.1000917

**Published:** 2010-04-22

**Authors:** Erika M. Wolff, Hyang-Min Byun, Han F. Han, Shikhar Sharma, Peter W. Nichols, Kimberly D. Siegmund, Allen S. Yang, Peter A. Jones, Gangning Liang

**Affiliations:** 1 Department of Urology, Norris Comprehensive Cancer Center, Keck School of Medicine, University of Southern California, Los Angeles, California, United States of America; 2 Department of Hematology, Norris Comprehensive Cancer Center, Keck School of Medicine, University of Southern California, Los Angeles, California, United States of America; 3 Department of Pharmacology and Pharmaceutical Sciences, Norris Comprehensive Cancer Center, Keck School of Medicine, University of Southern California, Los Angeles, California, United States of America; 4 Department of Pathology, Norris Comprehensive Cancer Center, Keck School of Medicine, University of Southern California, Los Angeles, California, United States of America; 5 Department of Preventive Medicine, Norris Comprehensive Cancer Center, Keck School of Medicine, University of Southern California, Los Angeles, California, United States of America; Ludwig Institute for Cancer Research, University of California San Diego, United States of America

## Abstract

It was recently shown that a large portion of the human transcriptome can originate from within repetitive elements, leading to ectopic expression of protein-coding genes. However the mechanism of transcriptional activation of repetitive elements has not been definitively elucidated. For the first time, we directly demonstrate that hypomethylation of retrotransposons can cause altered gene expression in humans. We also reveal that active LINE-1s switch from a tetranucleosome to dinucleosome structure, acquiring H2A.Z- and nucleosome-free regions upstream of TSSs, previously shown only at active single-copy genes. Hypomethylation of a specific LINE-1 promoter was also found to induce an alternate transcript of the *MET* oncogene in bladder tumors and across the entire urothelium of tumor-bearing bladders. These data show that, in addition to contributing to chromosomal instability, hypomethylation of LINE-1s can alter the functional transcriptome and plays a role not only in human disease but also in disease predisposition.

## Author Summary

A surprisingly large portion of our transcriptome originates within repetitive elements, most commonly LINE-1s. However, the mechanism of activation has not been definitively shown. We directly demonstrate for the first time the causal relationship between DNA hypomethylation and transcriptional activation of LINE-1 promoters. Hypomethylation of specific LINE-1 promoters can alter the transcriptome, including activating an alternate transcript of the *MET* oncogene, not only in primary bladder tumors but also in premalignant urothelium across entire bladders with tumors. Our study has important implications for tumor biology, cancer detection, and treatment, and it also answers the long-standing question of whether hypomethylation of retrotransposons induces ectopic gene expression and influences disease susceptibility in humans, a phenomenon first described in *agouti* mice.

## Introduction

Aberrant DNA methylation is involved in the initiation and progression of carcinogenesis and includes both hypermethylation of CpG islands at gene promoters and global hypomethylation. While a small portion of hypomethylation occurs at gene promoters, resulting in overexpression of certain oncogenes [1], [2], the majority occurs at repetitive elements, such as long interspersed nuclear elements (LINE-1s or L1s) [3]. Since most of the 500,000 copies of L1 have become nonfunctional over the course of human evolution [4] and can no longer transpose, genome-wide hypomethylation at L1s during tumorigenesis is thought to contribute mainly to chromosomal instability [5]. In mice hypomethylation of transposable elements can lead to disruption of normal gene function [6]. Viable yellow agouti (*A^vy^*) mice have a retrotransposon inserted into one allele of the *agouti* locus and when this retrotransposon is hypomethylated, which can occur *in utero* by limiting the maternal intake of methyl donors, it acts as an alternate promoter for *agouti*. Ectopic induction of the *agouti* gene results in altered coat color, obesity, and an increased incidence of tumors [6]. While it is well known that repetitive elements are hypomethylated in cancer, it has never been directly demonstrated that hypomethylation of a retrotransposon leads to ectopic gene expression in humans.

A recent study has revealed that more than 30% of transcription start sites in the human genome are located within repetitive elements, with just over 7% in L1s [7]. A full length L1 sequence (6 Kb) has a sense promoter driving transcription of its two open reading frames and an antisense promoter driving transcription in the opposite direction that can act as an alternate promoter for surrounding genes [8]–[10]. Almost 500 of these retrotransposons can induce ectopic gene expression in embryonic and cancerous tissues, revealing their potential role during both development and tumorigenesis [7]. However this study did not address the potential mechanism of how repetitive elements become transcriptionally active. Since the L1 promoter is a CpG island and methylated in normal somatic tissues it seems likely that epigenetic mechanisms are involved in its transcriptional silencing. There are many layers of epigenetic regulation responsible for regulating expression of single copy genes, including DNA methylation, histone modifications, and nucleosome occupancy [11]. While it is known that unmethylated retrotransposons in *Arabidopsis* [12] acquire the active histone variant H2A.Z, the chromatin structure in humans of repetitive elements, particularly active ones, has been largely ignored.

Until recently it has not been possible to study the promoters of individual L1s since the sequences are too similar to design primers for one particular locus [13]–[15]. Therefore a direct correlation between the epigenetic status of a specific L1 and expression of its associated transcript has not been possible. For the first time to our knowledge, we have elucidated the role of epigenetics in the transcriptional activity of L1s by utilizing novel assays capable of examining the methylation status and chromatin structure of specific L1s and expression of alternate transcripts originating from the L1 promoters. In addition to L1s being hypomethylated and transcriptionally active in bladder tumors we also found that a specific L1 located within the *MET* oncogene is active across entire bladders with cancer. The clinical implication of our finding is that surgical excision of the tumor would leave behind large areas of the bladder that remain epigenetically altered and express a potential oncogene. We also provide evidence that an active L1 acquires H2A.Z and nucleosome free regions upstream of TSSs, which has only been described previously at single copy genes, and undergoes chromatin remodeling from an inactive tetranucleosomal structure to an active dinucleosomal structure.

## Results

### Hypomethylation of specific L1s correlates with expression of alternate gene transcripts

To elucidate the mechanism of transcriptional activation of repetitive elements we used the sequence of the functional promoter of L1s to identify specific promoters potentially capable of expressing alternate transcripts of host genes. Figure S1 contains the genomic locations of the L1s, all of which are in an antisense orientation to the host gene allowing for transcripts in sense orientation to the gene's coding sequence. Interestingly, most these ESTs are from tumor cells. One such L1 is located within the *MET* oncogene (L1-*MET*) [8]. Since MET is known to be overexpressed in bladder cancer [16]–[18], we characterized two L1-*MET* transcripts by sequencing EST clones obtained from a bladder carcinoma cell line (GenBank accession no. BF208095) and placenta (BX334980). Both transcripts have start sites located in the L1 promoter, share the same reading frame as *MET* (Figure S2A), and when transiently transfected into Hela cells result in expression of truncated MET proteins (Figure S2B). Several truncated forms of the tyrosine kinase MET, which is the hepatocyte growth factor (HGF) receptor, are constitutively active and promote invasion and migration through activation of a variety of signal transduction pathways in numerous types of carcinomas, including breast, prostate, colorectal, and lung, in musculoskeletal sarcomas, and also in haematopoietic malignancies [19], [20]. Therefore hypomethylation of L1-*MET* could lead to expression of a transcript that encodes a truncated and potentially constitutively active MET protein.

**Figure 1:**
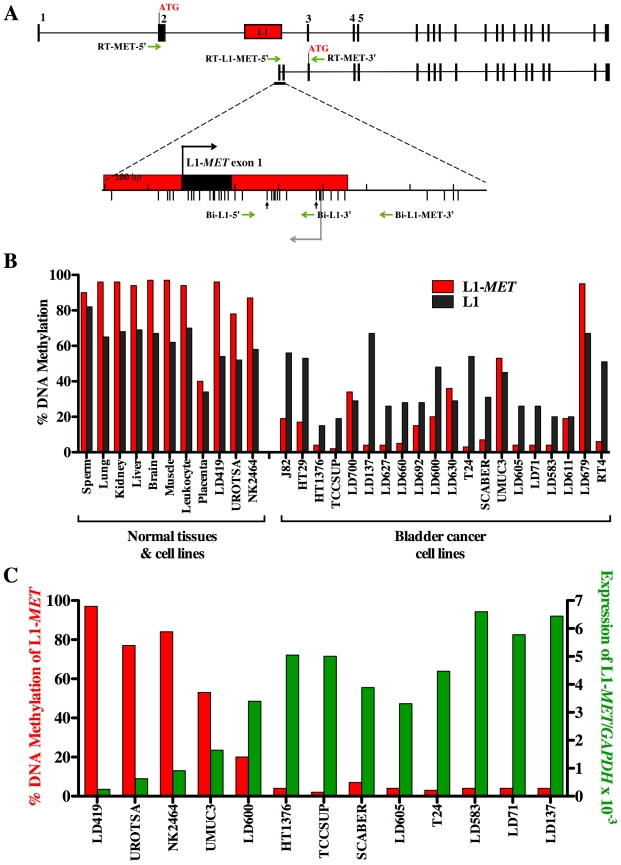
Methylation and expression of L1-*MET* correlates in cell lines. (A) Map of alternate transcript from L1-*MET*. Exons are represented by black boxes and a red box represents the specific L1. The bent arrows indicate transcriptional start sites and ATGs indicate translational start sites. Horizontal arrows indicate the primers for PCR of bisulfite converted DNA and RT–PCR. The bisulfite-specific primers Bi-L1-5′ and Bi-MET-3′ were used to amplify L1-*MET* for methylation analysis and Bi-L1-5′ and Bi-L1-3′ for global L1 methylation analysis. The RT–PCR primers, RT-L1-MET-5′ and RT-MET-3′ were used to amplify cDNA of the L1-*MET* transcript for expression analysis and RT-MET-3′ and RT-MET-5′ for the full length *MET* expression analysis. The lower tick marks represent each CpG site. Vertical arrows indicate the CpG sites analyzed by the Ms-SNuPE assay. (B) L1-*MET* methylation (red bars) and L1 methylation (black bars) was analyzed by Ms-SNuPE in 8 normal tissues, one normal bladder fibroblast cell line (LD419), two non-tumorigenic urothelial cell lines (UROtsa and NK2426), and 20 bladder carcinoma cell lines. Values are the average of one CpG site for L1 and an average of two CpG sites for L1-*MET* from technical triplicates. Error bars represent the standard deviation. (C) Expression of L1-*MET* was measured using real-time RT PCR in one normal bladder fibroblast cell line, two normal urothelial cell lines and 10 bladder carcinoma cell lines. There is clearly a strong correlation between DNA methylation and expression in all 13 cell lines examined. Values are the average from technical duplicates. Red bars indicate the methylation status of L1-*MET*, which is also represented in (B), and green bars represent the level of expression relative to *GAPDH*.

To examine the methylation status at a specific L1 we designed bisulfite-specific PCR primers with one located in the L1 promoter and the other in the surrounding intronic region of the host gene (Figure 1A). The L1-*MET* promoter was highly methylated in normal cells and tissues, whereas 18 out of 20 of the bladder carcinoma cell lines showed significant hypomethylation (p<3.4×10^−10^) (Figure 1B). We also measured methylation of global L1s using the standard assay with two primers that anneal within the L1 promoter (Figure 1A). We found that hypomethylation of L1s was significant (p<6.4×10^−5^) but not as dramatic as L1-*MET* hypomethylation and that the methylation pattern can be quite different between global L1s and a specific L1, such as in the cell lines LD137, T24, and RT4 (Figure 1B). This result clearly shows that global L1 status does not represent the status at specific L1s.

The transcript from the L1-*MET* anti-sense promoter contains its own exons 1 and 2, referred to as L1-*MET* exon 1 and L1-*MET* exon 2 (Figure 1A). We designed RT–PCR primers with one primer located in either the *MET* exon 2 or the L1-*MET* exon 1 and one primer located in the shared exon 3 to examine the expression of the host gene *MET* and the alternate transcript from L1-*MET*, respectively (Figure 1A). We confirmed the transcription start site of L1-*MET* by 5′RACE in the T24 bladder carcinoma cell line (Figure S2C) in which the L1-*MET* promoter is completely unmethylated. The L1-*MET* transcript was lowly expressed in one bladder fibroblast cell line (LD419) and two non-tumorigenic urothelial cell lines, UROtsa [21] and NK2426 [22], and highly expressed in most bladder carcinoma cell lines (Figure 1C). L1-*MET* was also not expressed in normal tissues except for placenta (data not shown). Therefore L1-*MET* hypomethylation correlated with the expression of the alternate transcript (Figure 1C). Treatment of LD419 with the demethylating agent 5-aza-deoxycytidine lead to expression of L1-*MET*, suggesting that L1-*MET* is silenced by DNA methylation (Figure S2D). We also designed bisulfite-specific PCR primers and RT–PCR primers for two additional specific L1s from the list shown in Figure S1, which were randomly selected. One L1 was located within *ACVR1C*, a member of the TGF-Beta family able to induce apoptosis [23], and the other located in *RAB3IP*, and a protein whose exact function is unknown (Figure S3 and Figure S4). Hypomethylation of these specific L1s also correlated with expression of their associated alternate transcripts, suggesting that DNA methylation plays a role in transcriptional silencing of functional L1 promoters in general (Figure S3 and Figure S4).

### DNA methylation silences the L1-*MET* promoter

**Figure 2:**
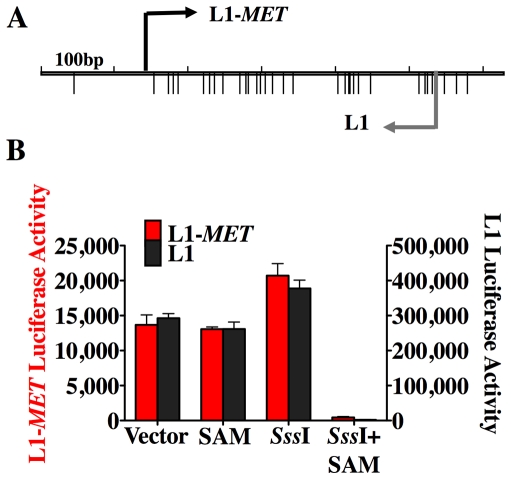
DNA methylation silences the L1-MET promoter. (A) Map of the CpG sites (represented by the lower tick marks) within the L1-*MET* anti-sense promoter (ch7:116364010–116364564), which was ligated into a CpG-less luciferase vector (pCpGL) in both orientations, allowing for the measurement of either L1-*MET* activity (red bars) or L1 activity (black bars). (B) The relative luciferase activity (firefly luciferase light units/Renilla luciverase light units) is represented as the mean ± SD and was high in the untreated vector, the methyl donor S-adenosyl-methionine (SAM) alone, and the CpG methyltransferase (SssI) alone. When the methyltransferase enzyme and the methyl donor (SssI+SAM) were added to the luciferase vectors together then promoter activity was silenced in both directions. The values are the average of three biological replicates. Error bars represent the standard deviation.

The data presented thus far represents an association between hypomethylation of an L1 promoter and ectopic expression of an alternate transcript. To directly demonstrate that DNA methylation represses transcription of the bidirectional L1 promoter we utilized a luciferase promoter activity assay with a pCpGL luciferase reporter construct that has been modified to not contain any CpG sites [24]. Therefore, after insertion of the promoter sequence of interest the plasmid can be treated with the CpG methyltransferase M. *Sss*I and the methyl donor S-adenosyl-methionine (SAM), allowing the promoter to be methylated without affecting the plasmid backbone. We created two plasmids, differing only the orientation of the L1-*MET* promoter, allowing us to measure either the L1 transcriptional activity or the L1-*MET* activity transcriptional activity (Figure 2A). Activity in both directions was inhibited in the methylated plasmid (Figure 2B). To our knowledge these data show for the first time that DNA methylation directly suppresses transcription from L1 promoter in both directions, indicating that the ectopic transcripts from L1s found in cancer [7] are a result of L1 hypomethylation. The relative activity between the two different promoters indicates that the L1-*MET* promoter is much weaker than the L1 promoter.

### Chromatin remodeling accompanies transcriptional activation of L1 promoters

**Figure 3:**
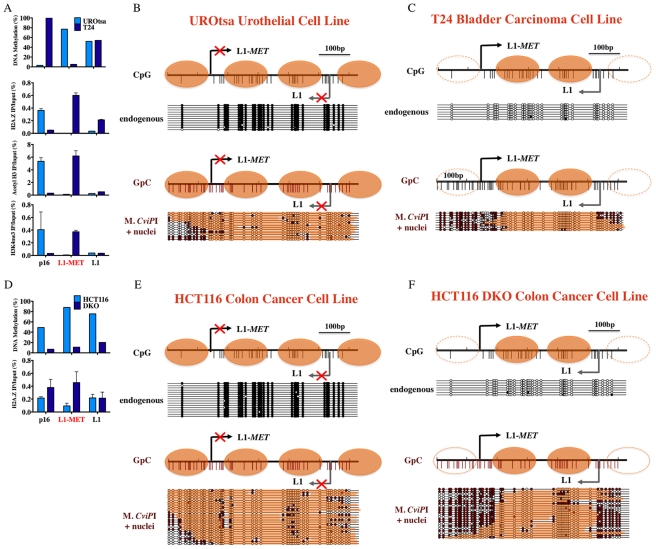
Chromatin remodeling occurs at an active L1-*MET* promoter. (A) DNA methylation at L1-*MET* and global L1s was determined by pyrosequencing in the immortalized urothelial cell line UROtsa and bladder carcinoma cell line T24. Chromatin immunoprecipitation was performed using antibodies for H3K4me3, acetylated H3, and H2A.Z. The values of the ChIP assay are the average of three experiments with technical duplicates. Error bars represent the standard deviation, and *p16* represents a single copy gene control. The presence of active histone marks was associated with absence of DNA methylation at L1-*MET* in the cancer cell line. Methylase dependent single promoter analysis (MSPA) with M. *CviP*I, a GpC methyltransferase, of the (B) endogenously methylated L1-*MET* promoter (ch7:116364020–116364664) in the UROtsa immortalized urothelial cell line and the (C) endogenously unmethylated L1-*MET* promoter in T24 bladder carcinoma cells. (D) DNA methylation at L1-*MET* and global L1s was determined by pyrosequencing in the colon cancer cell line HCT116 and HCT116 DKO cells (DNMT1 hypomorph/DNMT3B knockout) [31], [32]. Chromatin immunoprecipitation was performed using antibodies for H2A.Z. The presence of active histone marks was associated with absence of DNA methylation at L1-*MET* in the DKO cell line. Methylase dependent single promoter analysis (MSPA) with M. *CviP*I, a GpC methyltransferase, of the (E) endogenously methylated L1-*MET* promoter in HCT116 colon cancer cells, and (F) endogenously unmethylated L1-*MET* promoter in HCT116 DKO cells. White circles indicate unmethylated sites and black circles indicate methylated sites. Orange bars indicate areas of protection consistent with the presence of a nucleosome.

In addition to DNA methylation, epigenetic regulation of gene transcription also involves chromatin structure, specifically covalent modifications of histones, incorporation of histone variants, and nucleosome occupancy. In mice the chromatin structure of global L1s has been studied, but not in the promoter region [25]. Very few studies have addressed the chromatin structure at repetitive elements in humans. We took advantage of our ability to examine specific L1s to analyze the chromatin remodeling that occurs between the promoters of inactive and active repetitive elements in humans. Using chromatin immunoprecipitation (ChIP) we found that the level of DNA methylation at each specific L1 is inversely proportional to the level of enrichment of active histone marks (Figure 3A and Figure S5), and the chromatin structure at global L1s did not correlate with the specific L1s. Comparing the structure of the unmethylated L1-*MET* promoter in T24 bladder carcinoma cells to the methylated L1-*MET* promoter in UROtsa urothelial cells revealed a gain of the active marks H3K4me3 and acetylated H3 and the histone variant H2A.Z (Figure 3A). Therefore transcriptional activation of a repetitive element results in a similar pattern of chromatin remodeling found in active single copy genes such as p16 (Figure 3A) [12], [26], [27].

### A switch from a tetranucleosome to dinucleosome structure accompanies transcriptional activation of the L1-*MET* promoter

Methylase-sensitive Single Promoter Analysis (M-SPA) has previously been used to obtain single molecule resolution of nucleosome positioning at unmethylated CpG island promoters [28]. Briefly, nuclei are isolated and treated with the CpG methyltransferase M. *Sss*I, followed by DNA extraction, bisulfite conversion, and genomic sequencing of individual clones. The resulting pattern of applied DNA methylation reveals patches of protection, indicating the location of nucleosomes on individual molecules. Previously, the main limitation of the M-SPA method was that it could not be used to assess nucleosome positioning in an endogenously methylated region. However, the enzyme M. *CviP*I, which methylates GpC sites [29], can be used to avoid this problem since endogenous GpC sites are not methylated in humans except in the context of a GpCpG. Therefore, by modifying our M-SPA method by using a GpC methyltransferase we have conducted the first single molecule analysis of nucleosome positioning at a methylated promoter and, in combination with our ability to study specific L1s, have shown the nucleosome occupancy at a single repetitive element in both an active and inactive state.

The endogenously methylated L1-*MET* promoter in the UROtsa immortalized urothelial cell line was completely occupied by nucleosomes, revealing that the methylated L1-MET promoter exists in a tetranucleosomal structure (Figure 3B). GpCpG sites were excluded from analysis since it is not possible to distinguish between endogenous CpG methylation and enzyme-induced GpC methylation at such loci. When we performed the same assay on T24 cells in which L1-*MET* is unmethylated we found a nucleosome occupying the region downstream of each of the two transcription start sites and no nucleosome upstream of either (Figure 3C). We were able to confirm the results in T24 cells using the CpG methyltransferase M. *Sss*I, since L1-*MET* was not endogenously methylated (Figure S6). However, the number and location of CpG sites limits the resolution of this assay since the region upstream of the L1-*MET* start site contains only one CpG site. Therefore, the GpC methyltransferase allowed an increased resolution for this method. The unmethylated *MLH1* promoter was used as a positive control for both CpG and GpC methyltransferase activity and accessibility (data not shown).

Previous work on the *MLH1* bidirectional promoter has demonstrated that while each transcription start site loses the nucleosome directly upstream when active (−1 nucleosome), the nucleosome directly downstream is always maintained (+1 nucleosome) [27], [30]. The L1 promoter is a different type of bidirectional promoter that generates partially overlapping sense and antisense transcripts, commonly referred to as an antisense promoter (ASP). The L1 ASP has room for two nucleosomes between the two transcription start sites, therefore each start site has its own +1 nucleosome. These two +1 nucleosomes are maintained while the active promoter loses the −1 nucleosome at both starts sites. Therefore the inactive L1 promoter exists in a tetranucleosomal state (two +1 and two −1 nucleosomes) while the active promoter exists in a dinucleosomal state (two +1 nucleosomes). In addition, when DNA methylation levels are reduced by knocking out expression of 2 of the 3 methyltransferases responsible for maintaining DNA methylation, DNMT1 and DNMT3B [31], [32], we see acquisition of H2A.Z at L1-*MET* and global L1s (Figure 3D) along with induction of expression of L1-*MET* (data not shown) and nucleosome eviction at the L1-*MET* promoter (Figure 3E and 3F), revealing that a switch from an inactive tetranucleosomal structure to an active dinucleosomal structure accompanies hypomethylation.

### Many L1 promoters exist in an active chromatin structure

**Figure 4:**
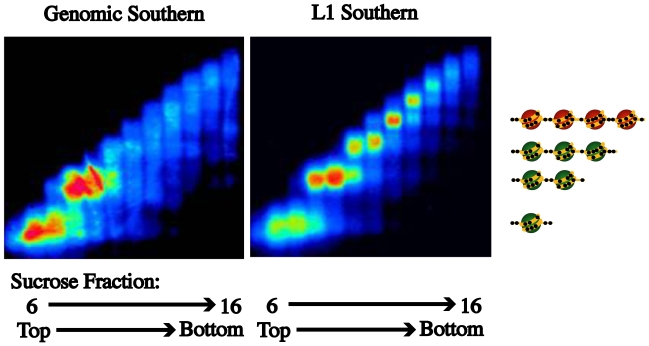
Nucleosome eviction is a frequent occurrence at L1 promoters. Partial MNase digestion of nucleosomes was followed by fractionation by a sucrose density gradient. When a Southern for genomic DNA was performed on the DNA in each fraction (6–16), enrichment in the mono- and dinucleosome fractions was revealed. When a Southern for L1s was performed enrichment of L1s in the di- and tetranucleosome fractions was found. According to our model the L1 promoters with a tetranucleosomal structure should be inactive and methylated.

While a single-molecule analysis of the nucleosome occupancy at the L1-*MET* promoter confirmed that an active L1 promoter switches from a tetranucleosomal structure to a dinucleosomal structure, we cannot generalize that other L1s exist in these states. To do so we took a cancer cell line that has a methylated and inactive L1-*MET* promoter, the colon cancer cell line HCT116, and performed chromatin fractionation using MNase digestion followed by sucrose gradient ultracentrifugation [33]. The fractions were run on an agarose gel and a genomic Southern using radioactively labeled input DNA was performed. Most of the DNA was present in the mononucleosome and dinucleosome fractions (Figure 4). When the same blot was probed with the L1 promoter sequence, the distribution of global L1 promoters showed enrichment in both the dinucleosome and tetranucleosome fractions, indicating that other L1s besides L1-*MET* could exist in an inactive tetranucleosome or active dinucleosome structure (Figure 4).

### Hypomethylation of and expression from specific L1s occurs in bladder tumors

**Figure 5:**
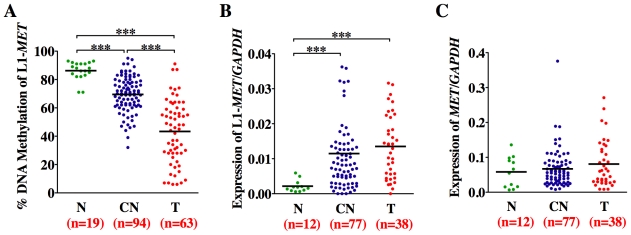
Methylation and expression status of L1-*MET* correlates in bladder tissues. Horizontal lines represent the mean and n the number of patient samples. (A) Methylation status was analyzed by Ms-SNuPE in normal tissues (N, green), corresponding normal tissues (CN, dark blue), and bladder tumors (T, red). Values are an average of two CpG sites. (B) Expression of the alternate transcript from L1-*MET* and (C) the host gene *MET*, and the control gene *GAPDH* was measured by real-time RT–PCR. *** represents p<0.001, ** represents p<0.01, and * represents p<0.05 as determined by the Mann-Whitney test. While there are no error bars for the clinical sample analysis due to the extremely limited amount of sample DNA, the results show a consistent trend.

Since bladder tumors display both hypomethylation of L1s [34] and overexpression of MET [16]–[18], our next step was to determine whether hypomethylation of the specific L1 promoters and their associated alternate transcripts, including L1-*MET*, were present in uncultured bladder tumors. We found high levels of methylation at L1-*MET* and low expression in normal bladder epithelium obtained from age-matched cancer free bladders (Figure 5A and 5B) and significant hypomethylation of, and expression from, L1-*MET* in bladder tumors (Figure 5A and 5B). We also examined the methylation and expression of two additional specific L1 promoters located within host genes (Figure S7). Hypomethylation of the L1-*ACVR1C* and L1-*RAB3IP* promoters occurred in bladder tumors (Figure S7). Therefore we have provided the first clinical evidence that hypomethylation of functional L1 promoters results in ectopic gene expression during tumorigenesis.

Surprisingly, we also found hypomethylation and associated alternate expression of L1-*MET* in the corresponding histologically normal tissues from tumor-bearing bladders taken at least 5 cm away from the tumor (p<0.0001) (Figure 5A and 5B). Hypomethylation and expression of L1-*MET* was more prevalent in the corresponding normal tissues than L1-*ACVR1C*, L1-*RAB3IP* (Figure S7) [35]. Therefore, hypomethylation of L1-*MET* and activation of alternate transcripts of *MET* occurs not only during tumorigenesis but also in premalignant tissue. Receiver operating characteristic (ROC) curves for L1-*MET* revealed an extraordinary degree of both sensitivity and specificity for detecting bladder tumors (AUC of 0.97) and premalignant tissue (AUCs of 0.89) (Figure S8). Since aberrant methylation in bladder tumors can be detected in urine sediments [36] and we are able to detect hypomethylation of L1-*MET* in urine sediments of bladder cancer patients (Figure S9), a noninvasive urine test has the potential to be developed into an assay for tumor detection and prediction of high-risk patients.

As expected, the expression of the host gene *MET* was not correlated with hypomethylation of the L1-*MET* promoter, since the expression of *MET* is regulated by its endogenous promoter and not by the specific L1 promoter (Figure 5A and 5C). It has previously been shown that overexpression of MET is correlated with global L1 hypomethylation in chronic myeloid leukemia (CML) [14]. The biological mechanism behind this correlation is unclear, as *MET* is expressed from an entirely different promoter than L1-*MET* and we have shown that global L1 methylation does not correlate with specific L1 methylation. Further, we did not find overexpression of *MET* in bladder tumors, suggesting that it may be L1-*MET* that is overexpressed instead since many primers used to detect expression can amplify both products.

### Hypomethylation and expression of L1-*MET* occurs across the urothelium of tumor-bearing bladders

**Figure 6:**
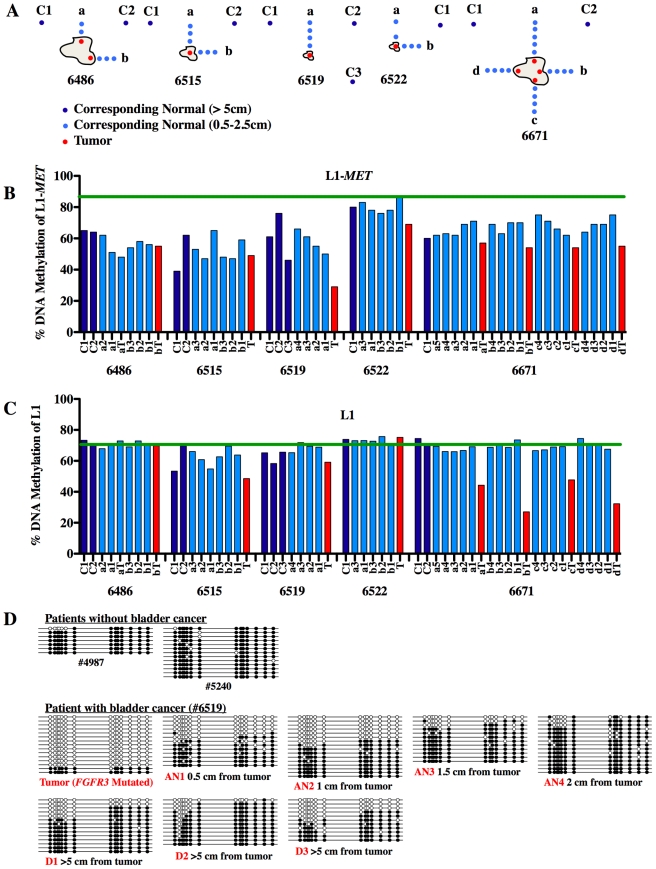
Methylation of L1-*MET* across the bladder. (A) Tissue samples were taken from five patients of their tumors (red, T) and at increasing distances from the tumor (0.5 to 2 cm) in the surrounding normal-appearing tissue in multiple directions (light blue, a to d). Additionally, distant normal-appearing samples were taken at least 5 cm from the tumor (dark blue, C). (B) Methylation at L1-*MET* and (C) global L1 was measured by pyrosequencing. The green line represents the mean methylation of normal samples from cancer-free patients. While there are no error bars for the clinical sample analysis due to the extremely limited amount of sample DNA, the results show a consistent trend. (D) Bisulfite sequencing of L1-*MET* was performed on samples from two bladder cancer-free patients (#4987 and #5240) and one bladder cancer patient (#6519). White circles represent unmethylated CpGs and black circles represent methylated CpGs.

Since we observed hypomethylation at L1-*MET* in bladder tissues taken at least 5 cm from tumors we collected histologically normal tissue samples from five tumor-bearing bladders taken at various distances and directions from the tumors to determine whether distance has any effect on the level of hypomethylation (Figure 6A). When compared to the average level of methylation in normal tissues from cancer-free bladders, L1-*MET* was dramatically hypomethylated in normal-appearing tissues across each of the tumor-bearing bladders independent of the distance from the site of the tumor (Figure 6B). However the normal-appearing tissues were not significantly hypomethylated at L1-*ACVR1C*, L1-*RAB3IP*, and global L1 (Figure S10 and Figure 6C). Bisulfite sequencing of L1-*MET* in the urothelium of patients without bladder cancer revealed only fully methylated strands while in a patient with bladder cancer fully unmethylated strands were present in the tumor and the corresponding normal urothelial tissue independent of the distance from the tumor (Figure 6D and Figure S11). A plot of the distribution of DNA strands versus the percent of methylated sites reveals a biphasic distribution in the patient with bladder cancer, with the majority of strands either fully methylated or fully unmethylated (Figure S11). Our *in vitro* results (Figure 2 and Figure 3) suggest that these fully unmethylated strands found in tumor-bearing bladders have undergone chromatin remodeling involving a switch from a tetranucleosome to a dinucleosome structure and are transcriptionally active. To our knowledge this is the first alteration, either epigenetic or genetic, that has been found across an entire tumor-bearing organ.

## Discussion

**Figure 7:**
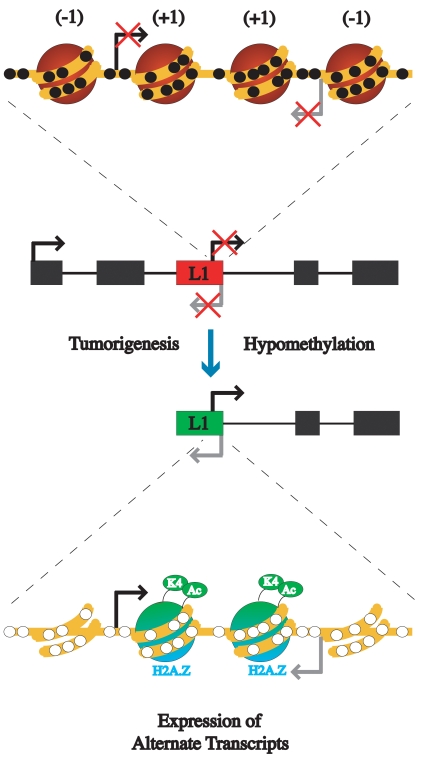
Model of the epigenetic alterations that occur between inactive L1s and active L1s during tumorigenesis. An L1 promoter is usually silenced by DNA methylation and has a compact chromatin structure with four nucleosomes occupying the promoter. Upon hypomethylation during tumorigenesis the L1 promoter becomes transcriptionally active. The active promoter loses a nucleosome upstream of each of the transcription start sites, resulting in a dinucleosome structure. The remaining nucleosomes have acetylated H3, H3K4me3, and H2A.Z. (−1) represents the nucleosome directly upstream of the transcription start site, while (+1) represents directly downstream nucleosome of transcriptional start site.

The consequences of global hypomethylation at repetitive elements in cancer has long been the subject of speculation regarding the generation of genomic instability and potential activation of oncogenes. Ever since studies on viable yellow agouti (*A^vy^*) mice revealed that hypomethylation of a retrotransposon could induce ectopic expression of a gene and influence disease susceptibility [6] it has been postulated that similar events may occur in humans. While hypomethylation during tumorigenesis occurs quite frequently, a direct demonstration of the impact of hypomethylation of repetitive elements on gene expression has not been conducted. Transcriptome sequencing has recently revealed the prevalence of transcripts originating from alternate TSS within repetitive elements in humans, indicating a potential functional role of activated repeats in altering gene expression [7]. Active L1s were mostly found in embryonic and cancerous tissues, many of which result alternate transcripts of protein-coding genes. Using several specific L1s we have demonstrated the mechanism of transcriptional activation and, taken together with the results of Faulkner *et al*. [7], our results highlight the previously underappreciated impact of hypomethylation on ectopic gene expression, possibly contributing to tumorigenesis in a synergistic or cooperative manner (see model in Figure 7).

To elucidate the mechanism of transcriptional activation of repetitive elements, we compared the epigenetic alterations, including methylation status, histone modifications, and nucleosome positioning, that occur at a single copy of an L1 between a transcriptionally inactive and active state. Since current methods did not exist for such a study we employed several novel assays, including using primers able to amplify specific L1s, enabling methylation and ChIP assays to be performed on single copies, and a modification of the method for determining nucleosome positioning at a single molecule resolution, currently limited to unmethylated CpG islands, which allowed for the determination of nucleosome positioning in a methylated region. We were able to show that transcription from the L1 promoter is silenced by DNA methylation, providing direct evidence that one function of DNA methylation is to protect the human genome from retrotransposons.

Transcriptional activation of L1 promoters by hypomethylation results in a chromatin structure similar to that of active single copy genes such as p16, revealing that the features of active promoters, such as acquisition of active histone marks, H2A.Z, and nucleosome free regions upstream of TSSs, are not restricted to canonical gene promoters. In addition, we found that the unique structure of the L1 promoter results in two very stable nucleosome occupancy states, the inactive tetranucleosome structure and the active dinucleosome structure, and that hypomethylation could result in a switch between the two. It has been demonstrated that tetranucleosomes form a compact chromatin fiber [37]. Therefore, the widespread chromatin remodeling due to global hypomethylation of L1 promoters could contribute to chromosomal instability through the loss of many stabilizing tetranucleosome structures.

To our knowledge we have provided the first direct evidence that transcriptional activation of repetitive elements is caused by hypomethylation and chromatin remodeling at their promoters, occurs in a human diseased state, and may play a role in disease predisposition. Specifically, hypomethylation of a L1 promoter induces an alternate transcript of the *MET* oncogene in bladder tumors and across the entire urothelium of tumor-bearing bladders. The presence of L1-*MET* hypomethylation across the entire urothelium of tumor-bearing bladders has several possible explanations. Epigenetic alterations such as hypermethylation of tumor suppressor genes and hypomethylation of L1s have been found in normal epithelia adjacent to several types of tumors, including breast [38], esophageal [39], and colon [40], [41], indicating the presence of a “field defect”. Our data supports the presence of an epigenetic field defect in bladders with cancer, either due to independent events across the urothelium or clonal expansion [42]. However, another possible explanation is that the loss of L1-*MET* methylation occurred during early development before the bladder was fully formed. While some evidence for such abnormal epigenetic programming exists, as a recent study revealed that people who develop bladder cancer have slightly lower levels of global DNA methylation in their blood than healthy control cases [43], we did not find any evidence of a loss of methylation at global L1s or specific L1s in our patient WBC samples (Figure S9). Another possibility, which cannot be ruled out by this data, is that the presence of a tumor causes epigenetic changes across the bladder.

Whatever the underlying mechanism, the modulation of gene expression by hypomethylation of a retrotransposon such as what occurs at the *agouti* locus in mice is also found in humans. This leads to the activation of surrounding genes, which may contribute to tumorigenesis in a synergistic or cooperative manner. Transurethral resection of bladder tumors would leave behind large areas of epigenetically altered urothelium, possibly contributing to the high level of recurrence of bladder cancer. Fortunately, hypomethylation at specific L1s seems to provide a valuable biomarker that has the potential to significantly impact the diagnosis and treatment of bladder cancer.

## Materials and Methods

### Cell lines

The non-tumorigenic human urothelial cell lines UROtsa and NK2426 and the normal fibroblast cell line LD419 have been described previously [21], [22], [36]. Human bladder carcinoma cell lines were obtained commercially (T24, J82, HT1376, SCaBER, UM-UC-3, TCCSUP, and RT4; American Type Culture Collection, Manassas, VA) or derived in our laboratory (prefix LD). Cell culture, DNA and RNA purification were performed as previously described [36]. RNA was reverse-transcribed as previously described [36]. 5′-Rapid Amplification of cDNA Ends (RACE) to determine the 5′ end of the primary transcript of L1-*MET* was performed using the RLM-RACE kit (Ambion) according to the manufacturer's instruction. See Table S1 for primer sequences.

### Tissue collection

Tumor tissue samples were collected from the patients undergoing cystectomy or TURBT for bladder cancer. Normal bladder epithelium was obtained from 12 patients undergoing radical prostatectomy for prostate cancer (aged from 50 to 80) and 7 autopsy patients aged from 34 to 82, 5 of which were from non-cancer related deaths and 2 from deaths due to cancers other than bladder). All of these collections took place at Norris Cancer Hospital in IRB-approved protocols with patients' consent. Hematoxylin and eosin (H&E) sections marked with the location of the adjacent urothelium or tumor were used to guide in microdissection. DNA was bisulfite treated as previously described [44]. RNA extraction was done using a RNAeasy Micro Kit (Qiagen, Crawley, UK).

### Quantitation of DNA methylation

Methylation-sensitive single nucleotide primer extention (MS-SNuPE) was performed as previously described [44]. See Table S1 for primer sequences. In order to allow for a higher throughput in methylation analysis pyrosequencing was also performed as described previously [45]. Testing both methods on the same set of 66 samples yielded a correlation in the methylation levels of R = 0.91 (Figure S12). For pyrosequencing, PCR was performed on bisulfite converted DNA using a biotin-labeled 3′ primer to enable purification and denaturation of the product by Streptavidin Sepharose beads and was followed by annealing of a sequencing primer to the single-stranded PCR product. Pyrosequencing was performed using the PSQ HS96 Pyrosequencing System and the degree of methylation was expressed for each DNA locus as percentage methylated cytosines over the sum of methylated and unmethylated cytosines. See Table S1 for primer sequences. To analyze the methylation status of individual DNA molecules, we cloned bisulfite PCR fragments into the pCR2.1 vector using the TOPO-TA cloning kit (Invitrogen, Carlsbad, CA). Individual colonies were screened for the insert and the region of interest was sequenced using M13 primers. See Table S1 for primer sequences.

### Quantitative RT–PCR

Expression was determined by quantitative RT–PCR as described previously [27]. See Table S1 for primer sequences.

### Luciferase assay

The L1-*MET* and L1 promoters were cloned into the pCpGL luciferase vector [24]. The portion of the L1-*MET* promoter cloned was 555 bp, with 535 bp within the L1 and 20 bp within the *MET* gene (ch7:116364010–564). These experiments were performed as described previously [24].

### Chromatin immunoprecipitation

ChIP was performed as described previously [27]. Briefly, chromatin was isolated from cells and crosslinked with formaldehyde. The chromatin was then sonicated to less than 500 bp in length and immunoprecipitated with an antibody to the histone modification of interest. Enrichment was determined by RT–PCR of the pulled down DNA. See Table S1 for primer sequences.

### Methylation-dependent single promoter analysis

M-SPA was performed as described previously [28]. Briefly, chromatin was isolated from 250,000 cells and treated for 15 minutes with 50 U of M. *Sss*I. DNA was isolated, bisulfite converted, and PCR fragments were cloned for sequencing of individual molecules. In order to examine endogenously methylated promoters and increase the resolution of this method, chromatin from 250,000 cells was treated with the enzyme M. *CviP*I, which methylates GpC sites [29], for 15 minutes with 100 U.

### MNase digestion and Southern blot

MNase digestion and sucrose density gradient centrifugation were performed as described previously [33]. See Table S1 for primer sequences for the LINE-1 promoter probe.

### Statistical analyses

Significant differences in methylation and expression levels in normal, corresponding normal, and tumor tissues were determined using a Mann-Whitney test.

## Supporting Information

Figure S1 Specific L1s with alternate transcripts located in intron of genes. Black boxes represent exons of the host gene while red boxes represent a specific L1. The black arrow represents the transcriptional start site of the host gene while the red arrow represents the alternate transcriptional start site within the potentially active L1 promoter. GenBank accession numbers for representative alternate transcripts are followed by the number in parentheses of similar transcripts transcribed from the individual L1. All L1s are antisense to their host genes, yielding alternate transcripts that are sense with their host genes.
(0.56 MB TIF)

Figure S2 The truncated MET protein encoded by L1-*MET*. (A) The functional domains of MET include the signal peptide (SP), sema domain at the N-terminus, the PSI domain, IPT repeats, the transmembrane domain (TM), and the kinase domain at the C-terminus. The structure of truncated MET proteins 1 and 2 are shown, encoded by transcripts derived from placenta (GenBank accession no. BX334980) and a bladder carcinoma cell line (BF208095), respectively. (B) The two L1-*MET* transcripts, truncated L1-*MET*-1 (T-*MET*-1) and truncated L1-*MET*-2 (T-*MET*-2), were cloned into a pMEV expression vector with 2 HA tags fused at the N-terminal. Hela cells were transfected with either the empty pMEV vector, pMEV T-*MET*-1, or pMEV T-*MET*-2 and protein was extracted after 48 hours. The expression of truncated MET-1 (90 kDa) and truncated MET-2 (60 kDa) was detected by western blot using an HA antibody. (C) Results of 5′RACE reveal the start site for L1-MET within the L1 element. The transcriptional start site of L1-*MET* was confirmed by 5′RACE in the T24 cell line which expressed L1-MET. The underlined sequence is located inside of the LINE-1. (D) RT–PCR analysis of reactivation of L1-MET by 1 or 3 µM of 5-Aza-CdR treatment for 24 hours (day 3 after treatment). β-actin expression level was used as a control.
(1.22 MB TIF)

Figure S3 Methylation and expression of L1-*ACVR1C* correlates in cell lines. (A) Map of alternate transcripts from L1-*ACVR1C*. Exons are represented by black boxes while the specific L1s are represented by red boxes. The lower tick marks represent each CpG site. The left bent arrow indicates transcriptional start sites and ATGs indicate translational start sites. Green arrows indicate the primers used to amplify the pyrosequencing product and the black arrow in between indicates the location of the pyrosequencing primer for L1-*ACVR1C*. (B) L1-*ACVR1C* methylation (red bars) and L1 methylation (black bars) was analyzed by pyrosequencing in 6 normal tissues, one normal bladder fibroblast cell line (LD419), one non-tumorigenic urothelial cell lines (UROtsa), and 10 bladder carcinoma cell lines. Values are the average of one CpG site for L1 and an average of two CpG sites for L1-*ACVR1C* from two technical duplicates. (C) Expression of L1- *ACVR1C* was measured using real-time RT PCR in one normal bladder fibroblast cell line, one normal urothelial cell line, and 10 bladder carcinoma cell lines. Values are also the average from two technical duplicates. Red bars indicate the methylation status of L1-*ACVR1C*, which is also represented in (B), and green bars represent the level of expression relative to *GAPDH*.
(0.86 MB TIF)

Figure S4 Methylation and expression of L1-*RAB3IP* correlates in cell lines. (A) Map of alternate transcripts from L1-*RAB3IP*. Exons are represented by black boxes while the specific L1s are represented by red boxes. The lower tick marks represent each CpG site. The left bent arrow indicates transcriptional start sites and ATGs indicate translational start sites. Green arrows indicate the primers used to amplify the pyrosequencing product and the black arrow in between indicates the location of the pyrosequencing primer for L1-*RAB3IP*. (B) L1-*RAB3IP* methylation (red bars) and L1 methylation (black bars) was analyzed by pyrosequencing in 6 normal tissues, one normal bladder fibroblast cell line (LD419), one non-tumorigenic urothelial cell lines (UROtsa), and 10 bladder carcinoma cell lines. Values are the average of one CpG site for L1 and an average of two CpG sites for L1-*RAB3IP* from two technical duplicates. (C) Expression of L1-*RAB3IP* was measured using real-time RT–PCR in one normal bladder fibroblast cell line, one normal urothelial cell line, and 10 bladder carcinoma cell lines. Values are also the average from two technical duplicates. Red bars indicate the methylation status of L1-*RAB3IP*, which is also represented in (B), and green bars represent the level of expression relative to *GAPDH*.
(0.88 MB TIF)

Figure S5 Chromatin remodeling occurs at active L1 promoters. (A) DNA methylation at specific and global L1s (with p16 as a control) was determined by pyrosequencing in the immortalized urothelial cell line UROtsa and bladder carcinoma cell line T24. The specific L1s had less methylation in the cancer cell line. Chromatin immunoprecipitation was performed using antibodies for (B) H3K4me3; (C) acetylated H3; and (D) H2A.Z. The values of the ChIP assay are the average of three experiments with technical duplicates. Error bars represent the standard deviation. The presence of active histone marks was associated with absence of DNA methylation at the specific L1s in the cancer cell line.
(0.67 MB TIF)

Figure S6 Chromatin remodeling occurs an active L1-*MET* promoter. Nucleosome positioning in an active fully unmethylated L1-*MET* promoter in T24 bladder carcinoma cells reveals a dinucleosomal structure, as determined by both M. *Sss*I, a CpG methyltransferase and M. *CviP*I accessibility.
(1.29 MB TIF)

Figure S7 Methylation and expression status of specific L1s correlates in bladder tissues. Horizontal lines represent the mean. (A) Methylation status of L1-*ACVR1C* was analyzed by pyrosequencing in normal tissues (N), corresponding normal tissues (CN), and bladder tumors (T). Values are an average of two CpG sites. (B) Expression of the alternate transcript from L1-*ACVR1C* and (C) the host gene *ACVR1C*, and the control gene *GAPDH* was measured by real-time RT–PCR. *** represents p<0.001, ** represents p<0.01, and * represents p<0.05. (D) Methylation status of L1-*RAB3IP* was analyzed by pyrosequencing in normal tissues (N), corresponding normal tissues (CN), and bladder tumors (T). Values are an average of two CpG sites. (E) Expression of the alternate transcript from L1-*RAB3IP* and F. the host gene *RAB3IP*, and the control gene *GAPDH* was measured by real-time RT–PCR. *** represents p<0.001, ** represents p<0.01, and * represents p<0.05 as determined by the Mann-Whitney test. While there are no error bars for the clinical sample analysis due to the extremely limited amount of sample DNA. the results show a consistent trend.
(0.58 MB TIF)

Figure S8 ROC curves for specific L1s. (A) ROC curves using L1-*MET* methylation distinguish between normal bladder tissue (N) and corresponding normal bladder tissues (CN), N and bladder tumors (T), and CN and T. (B) ROC curves using L1-*ACVR1C* methylation, and (C) ROC curves using L1-*RAB3IP* methylation. *** represents p<0.001 and * represents p<0.05.
(0.65 MB TIF)

Figure S9 Detection of L1-MET hypomethylation in urine sediments of patients with bladder cancer. Bisulfite-specific primers and a probe were designed for the MethyLight assay that amplified only completely unmethylated strands of L1-*MET*. Bladder tissues (N) from age-matched patients without bladder cancer (n = 10) and urine (N) from age-matched healthy volunteers (n = 10) showed low levels of L1-*MET* hypomethylation. However, urine (n = 20) from patients with TCC showed high levels of L1-*MET* hypomethylation, which was specific to the bladder since it was not detected in their white blood cells (WBC) (n = 20). Unmethylated levels (Y axis) indicate the Percent of fully Unmethylated Reference (PUR) values.
(0.30 MB TIF)

Figure S10 Methylation of specific L1s across the bladder. (A) Tissue samples were taken from five patients of their tumors (red, T) and at increasing distances from the tumor (0.5 to 2 cm) in the surrounding normal-appearing tissue in multiple directions (light blue, a to d). Additionally, distant normal-appearing samples were taken at least 5 cm from the tumor (dark blue, C). (B) Methylation at L1-*ACVR1C* and (C) L1-*RAB3IP* was measured by pyrosequencing. The green line represents the mean methylation of 12 normal samples from cancer-free patients. While there are no error bars for the clinical sample analysis due to the extremely limited amount of sample DNA. the results show a consistent trend.
(1.62 MB TIF)

Figure S11 Bisulfite sequencing of L1-*MET*. Biphasic distribution of L1-*MET* methylation status in corresponding tissue from a patient with bladder cancer is revealed by plotting the number of DNA strands by the percent of CpG sites methylated.
(0.18 MB TIF)

Figure S12 Ms-SNuPE and pyrosequencing yield similar methylation results. While both Ms-SNuPE and Pyrosequencing are quantitative assays, Pyrosequencing is much more high throughput. Therefore, we developed a Pyrosequencing assay for the rest studies. (A) We measured 4 CpG sites by Pyrosequencing assay in contrast with the 2 CpG sites by Ms-SNuPE. (B) We randomly chose 66 samples previously analyzed by Ms-SNuPE to perform Pyrosequencing on and the results are very similar from both assays (R = 0.91).
(0.42 MB TIF)

Table S1 Primer sequences.
(0.08 MB DOC)
