# Supplementary figures and images for: Hypomethylation of a LINE-1 Promoter Activates an Alternate Transcript of the MET Oncogene in Bladders with Cancer
Source: PLoS Genet. 2010 Apr 22;6(4):e1000917. doi: 10.1371/journal.pgen.1000917 (PMC2858672; doi:10.1371/journal.pgen.1000917)

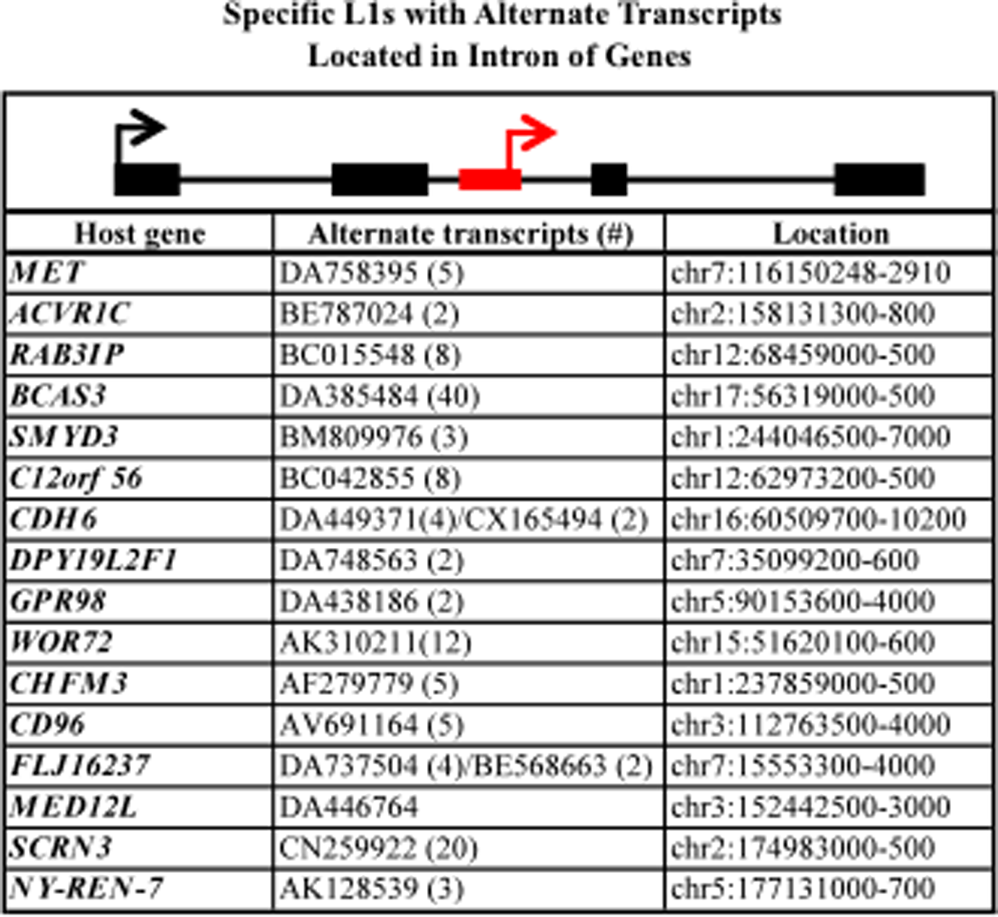

Supplement: Figure S1 — Specific L1s with alternate transcripts located in intron of genes. Black boxes represent exons of the host gene while red boxes represent a specific L1. The black arrow represents the transcriptional start site of the host gene while the red arrow represents the alternate transcriptional start site within the potentially active L1 promoter. GenBank accession numbers for representative alternate transcripts are followed by the number in parentheses of similar transcripts transcribed from the individual L1. All L1s are antisense to their host genes, yielding alternate transcripts that are sense with their host genes. (0.56 MB TIF) [file pgen.1000917.s001.tif]

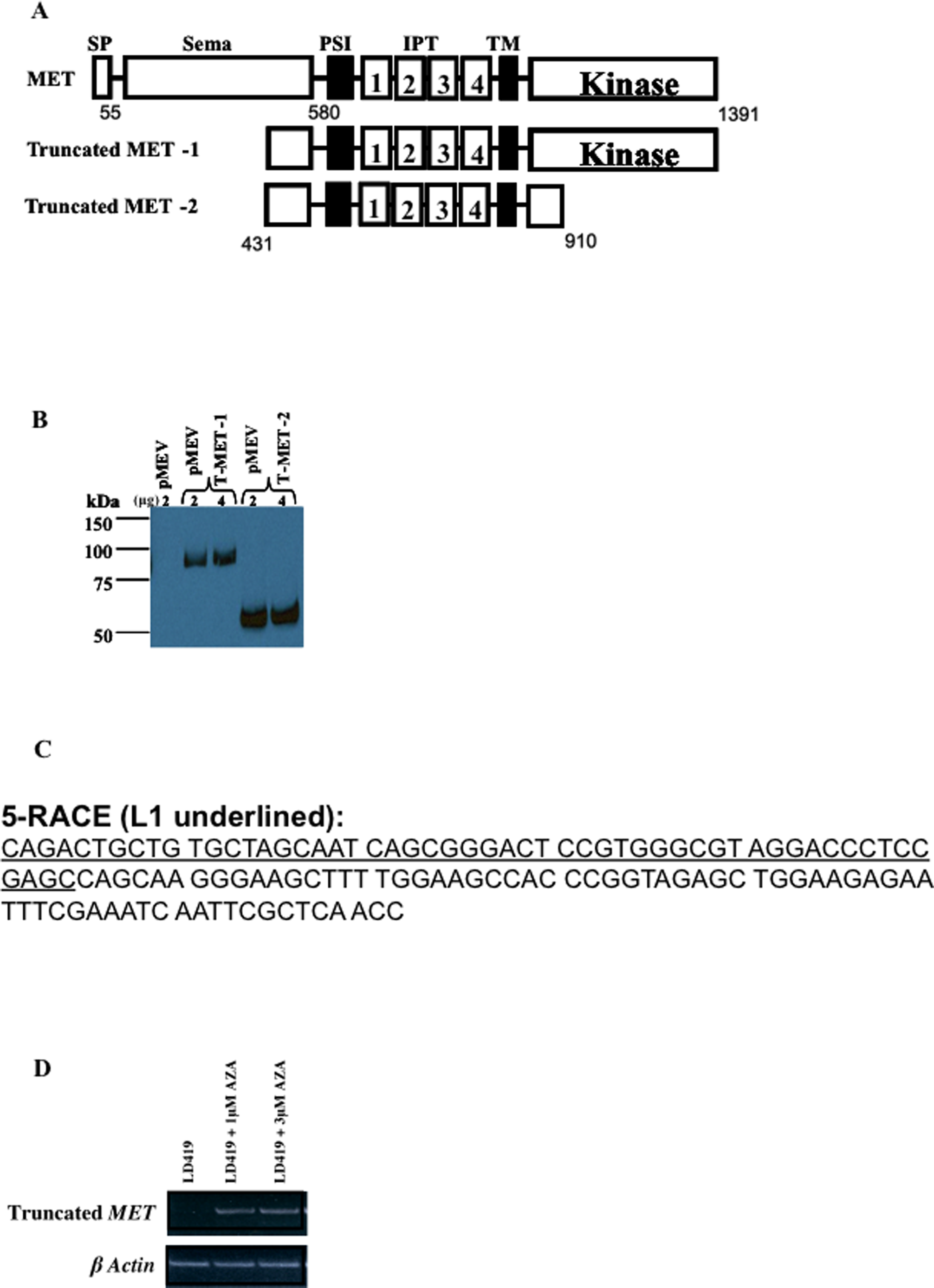

Supplement: Figure S2 — The truncated MET protein encoded by L1-MET. (A) The functional domains of MET include the signal peptide (SP), sema domain at the N-terminus, the PSI domain, IPT repeats, the transmembrane domain (TM), and the kinase domain at the C-terminus. The structure of truncated MET proteins 1 and 2 are shown, encoded by transcripts derived from placenta (GenBank accession no. BX334980) and a bladder carcinoma cell line (BF208095), respectively. (B) The two L1-MET transcripts, truncated L1-MET-1 (T-MET-1) and truncated L1-MET-2 (T-MET-2), were cloned into a pMEV expression vector with 2 HA tags fused at the N-terminal. Hela cells were transfected with either the empty pMEV vector, pMEV T-MET-1, or pMEV T-MET-2 and protein was extracted after 48 hours. The expression of truncated MET-1 (90 kDa) and truncated MET-2 (60 kDa) was detected by western blot using an HA antibody. (C) Results of 5′RACE reveal the start site for L1-MET within the L1 element. The transcriptional start site of L1-MET was confirmed by 5′RACE in the T24 cell line which expressed L1-MET. The underlined sequence is located inside of the LINE-1. (D) RT–PCR analysis of reactivation of L1-MET by 1 or 3 µM of 5-Aza-CdR treatment for 24 hours (day 3 after treatment). β-actin expression level was used as a control. (1.22 MB TIF) [file pgen.1000917.s002.tif]

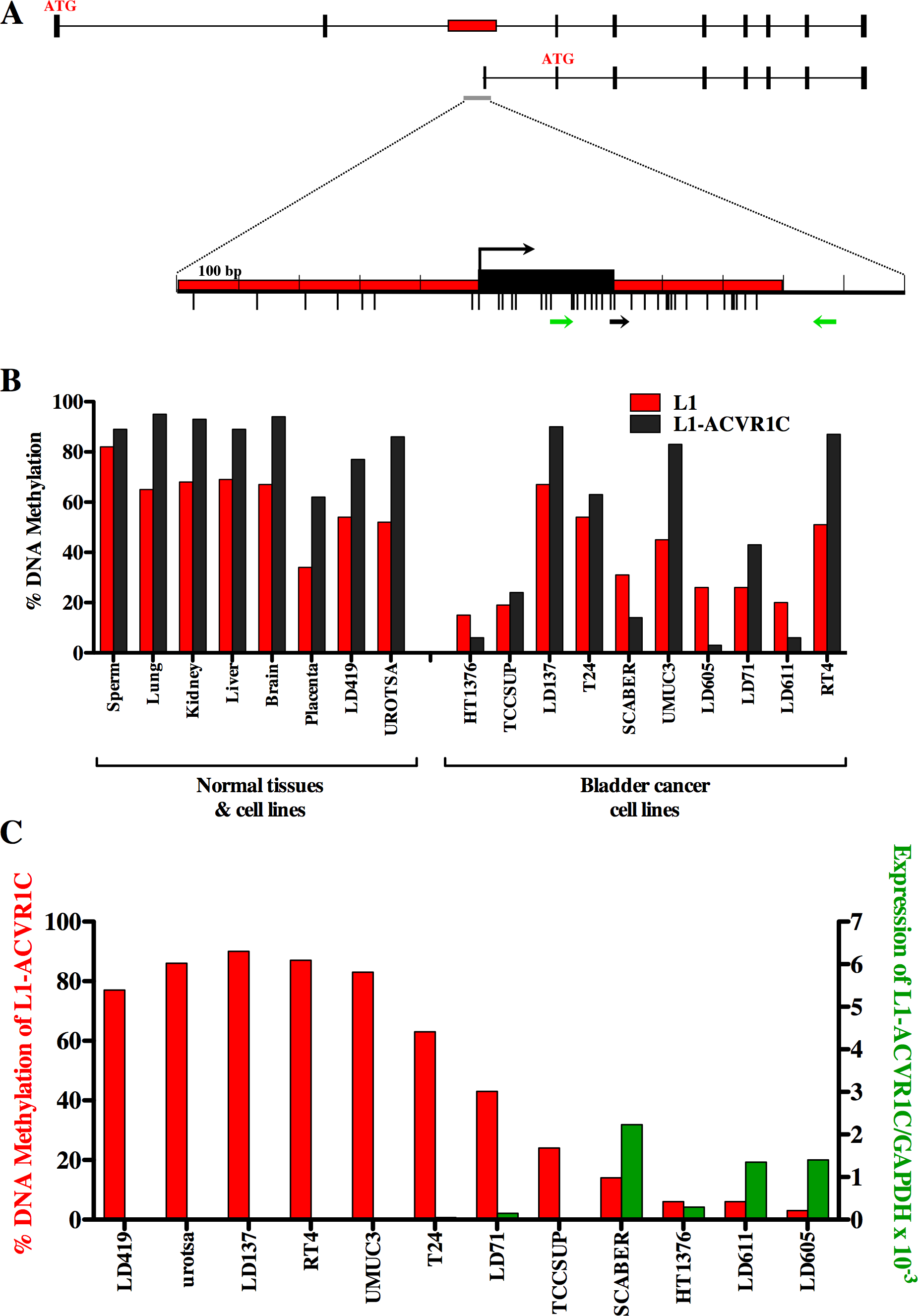

Supplement: Figure S3 — Methylation and expression of L1-ACVR1C correlates in cell lines. (A) Map of alternate transcripts from L1-ACVR1C. Exons are represented by black boxes while the specific L1s are represented by red boxes. The lower tick marks represent each CpG site. The left bent arrow indicates transcriptional start sites and ATGs indicate translational start sites. Green arrows indicate the primers used to amplify the pyrosequencing product and the black arrow in between indicates the location of the pyrosequencing primer for L1-ACVR1C. (B) L1-ACVR1C methylation (red bars) and L1 methylation (black bars) was analyzed by pyrosequencing in 6 normal tissues, one normal bladder fibroblast cell line (LD419), one non-tumorigenic urothelial cell lines (UROtsa), and 10 bladder carcinoma cell lines. Values are the average of one CpG site for L1 and an average of two CpG sites for L1-ACVR1C from two technical duplicates. (C) Expression of L1- ACVR1C was measured using real-time RT PCR in one normal bladder fibroblast cell line, one normal urothelial cell line, and 10 bladder carcinoma cell lines. Values are also the average from two technical duplicates. Red bars indicate the methylation status of L1-ACVR1C, which is also represented in (B), and green bars represent the level of expression relative to GAPDH. (0.86 MB TIF) [file pgen.1000917.s003.tif]

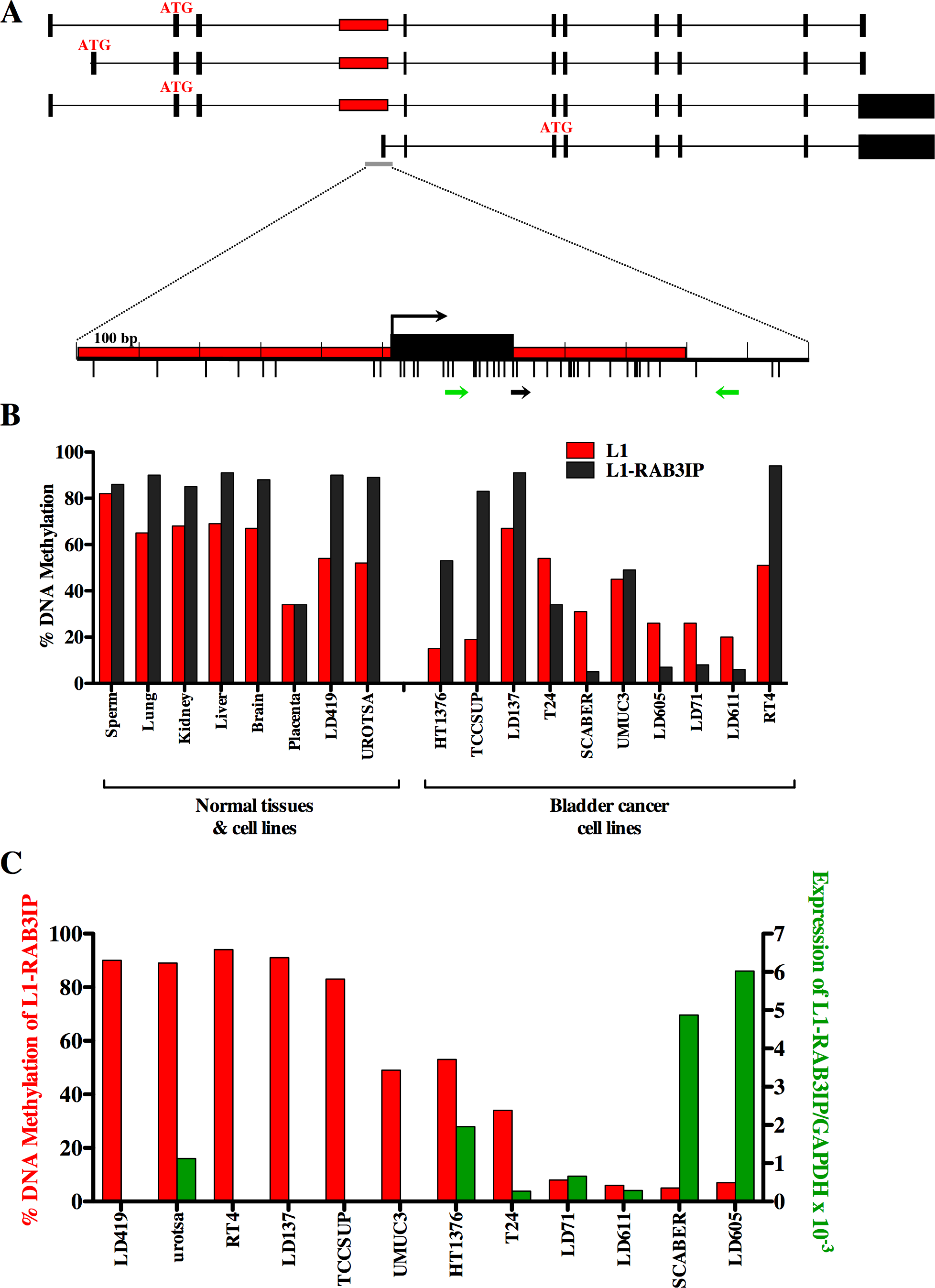

Supplement: Figure S4 — Methylation and expression of L1-RAB3IP correlates in cell lines. (A) Map of alternate transcripts from L1-RAB3IP. Exons are represented by black boxes while the specific L1s are represented by red boxes. The lower tick marks represent each CpG site. The left bent arrow indicates transcriptional start sites and ATGs indicate translational start sites. Green arrows indicate the primers used to amplify the pyrosequencing product and the black arrow in between indicates the location of the pyrosequencing primer for L1-RAB3IP. (B) L1-RAB3IP methylation (red bars) and L1 methylation (black bars) was analyzed by pyrosequencing in 6 normal tissues, one normal bladder fibroblast cell line (LD419), one non-tumorigenic urothelial cell lines (UROtsa), and 10 bladder carcinoma cell lines. Values are the average of one CpG site for L1 and an average of two CpG sites for L1-RAB3IP from two technical duplicates. (C) Expression of L1-RAB3IP was measured using real-time RT–PCR in one normal bladder fibroblast cell line, one normal urothelial cell line, and 10 bladder carcinoma cell lines. Values are also the average from two technical duplicates. Red bars indicate the methylation status of L1-RAB3IP, which is also represented in (B), and green bars represent the level of expression relative to GAPDH. (0.88 MB TIF) [file pgen.1000917.s004.tif]

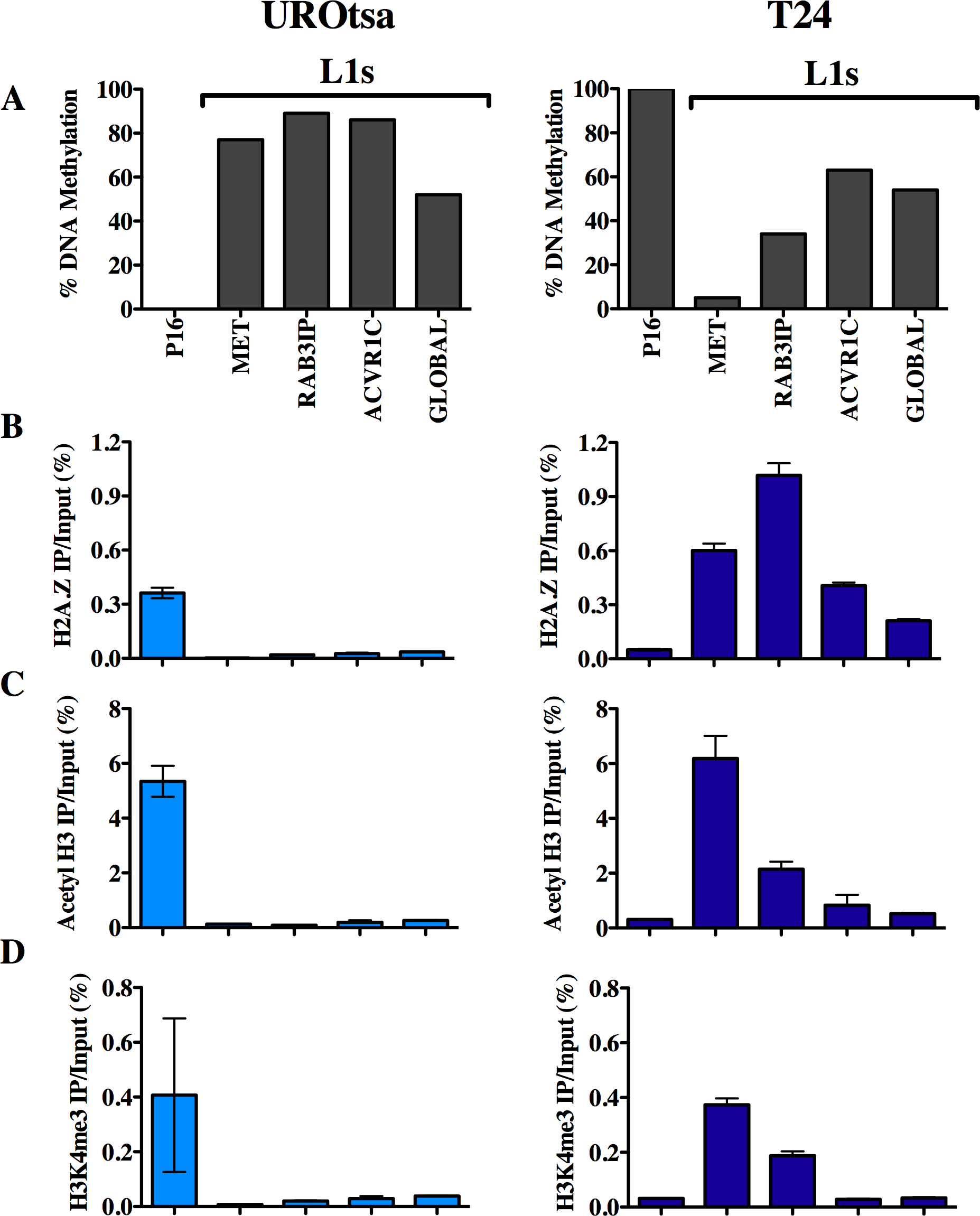

Supplement: Figure S5 — Chromatin remodeling occurs at active L1 promoters. (A) DNA methylation at specific and global L1s (with p16 as a control) was determined by pyrosequencing in the immortalized urothelial cell line UROtsa and bladder carcinoma cell line T24. The specific L1s had less methylation in the cancer cell line. Chromatin immunoprecipitation was performed using antibodies for (B) H3K4me3; (C) acetylated H3; and (D) H2A.Z. The values of the ChIP assay are the average of three experiments with technical duplicates. Error bars represent the standard deviation. The presence of active histone marks was associated with absence of DNA methylation at the specific L1s in the cancer cell line. (0.67 MB TIF) [file pgen.1000917.s005.tif]

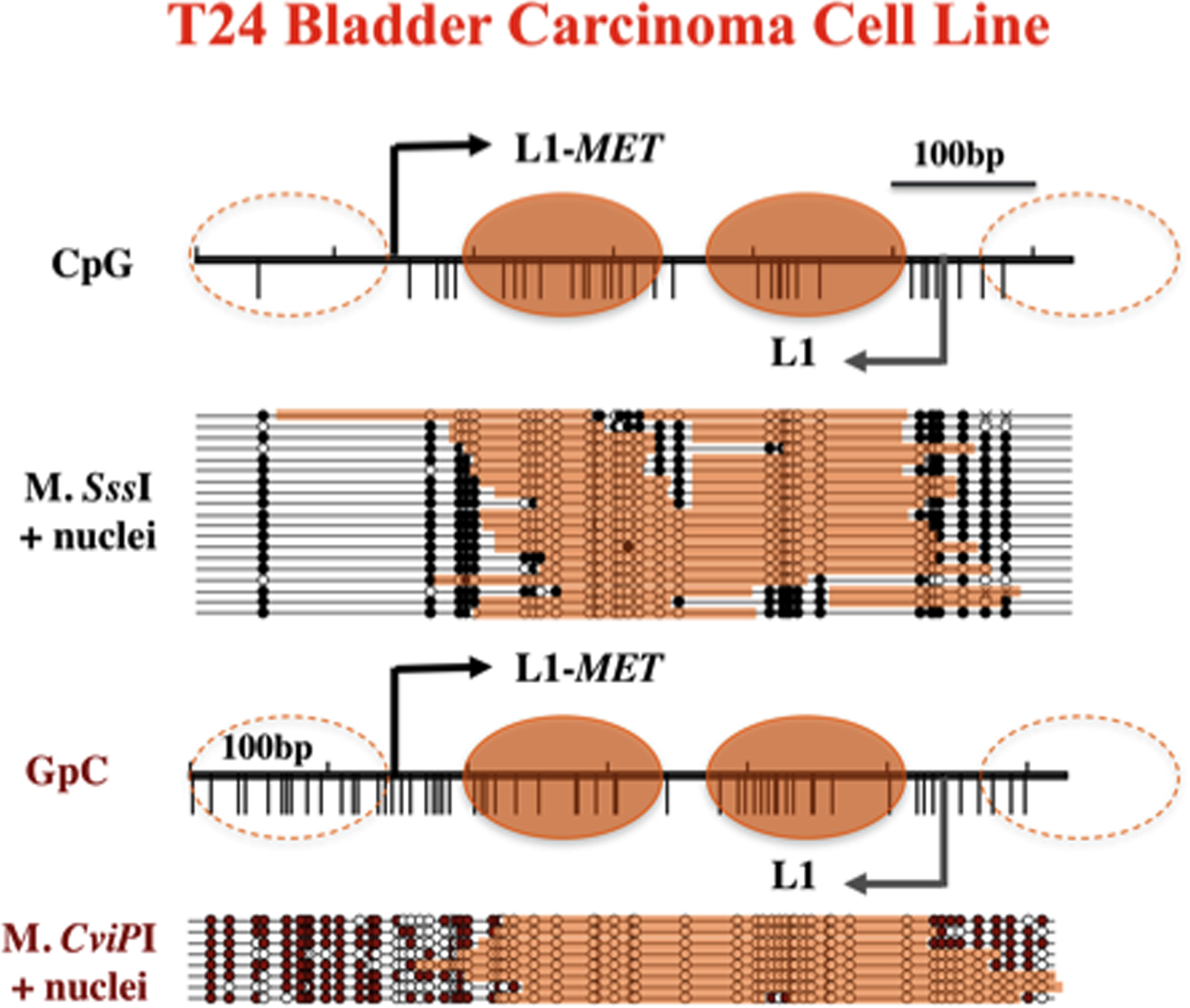

Supplement: Figure S6 — Chromatin remodeling occurs an active L1-MET promoter. Nucleosome positioning in an active fully unmethylated L1-MET promoter in T24 bladder carcinoma cells reveals a dinucleosomal structure, as determined by both M. SssI, a CpG methyltransferase and M. CviPI accessibility. (1.29 MB TIF) [file pgen.1000917.s006.tif]

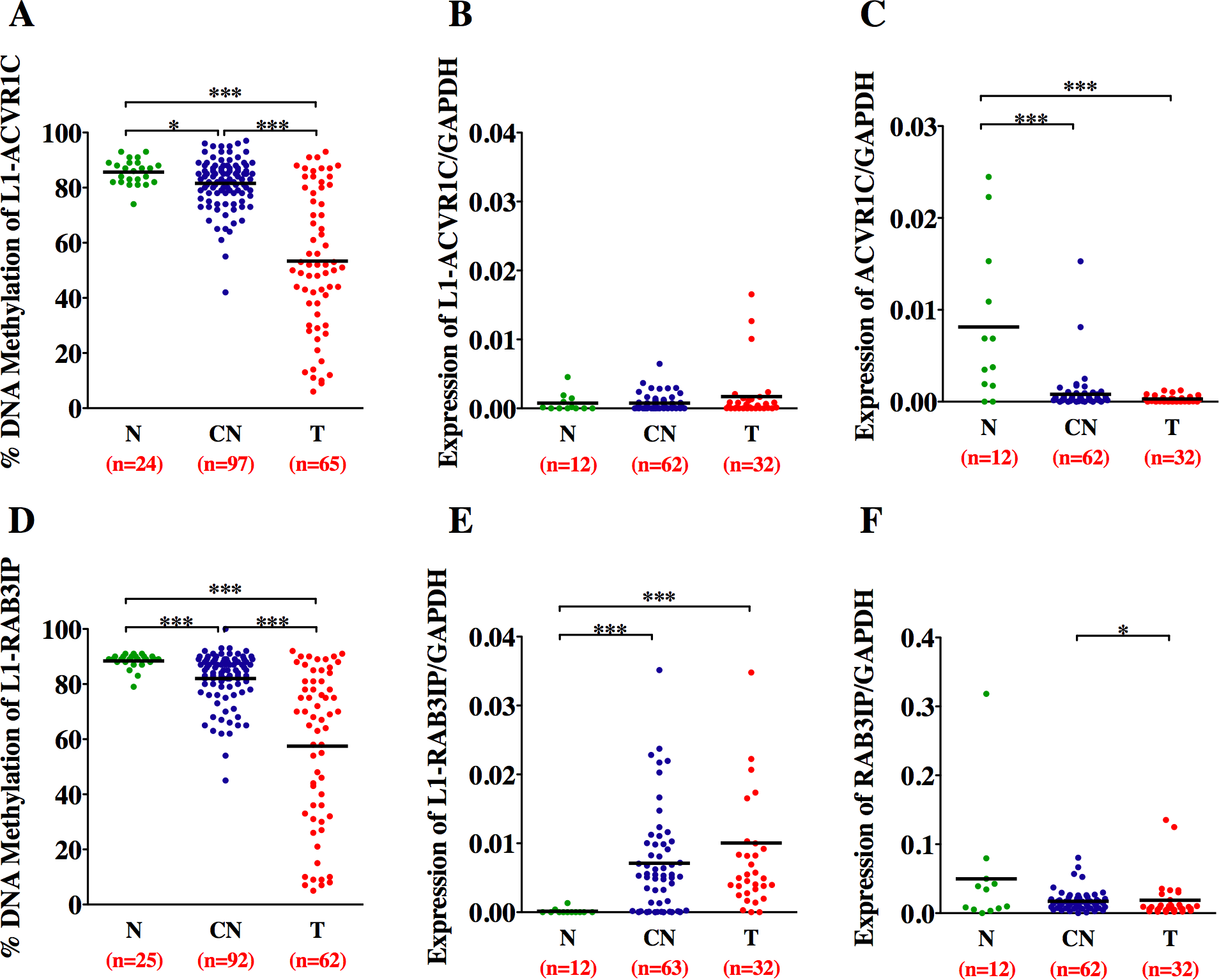

Supplement: Figure S7 — Methylation and expression status of specific L1s correlates in bladder tissues. Horizontal lines represent the mean. (A) Methylation status of L1-ACVR1C was analyzed by pyrosequencing in normal tissues (N), corresponding normal tissues (CN), and bladder tumors (T). Values are an average of two CpG sites. (B) Expression of the alternate transcript from L1-ACVR1C and (C) the host gene ACVR1C, and the control gene GAPDH was measured by real-time RT–PCR. *** represents p<0.001, ** represents p<0.01, and * represents p<0.05. (D) Methylation status of L1-RAB3IP was analyzed by pyrosequencing in normal tissues (N), corresponding normal tissues (CN), and bladder tumors (T). Values are an average of two CpG sites. (E) Expression of the alternate transcript from L1-RAB3IP and F. the host gene RAB3IP, and the control gene GAPDH was measured by real-time RT–PCR. *** represents p<0.001, ** represents p<0.01, and * represents p<0.05 as determined by the Mann-Whitney test. While there are no error bars for the clinical sample analysis due to the extremely limited amount of sample DNA. the results show a consistent trend. (0.58 MB TIF) [file pgen.1000917.s007.tif]

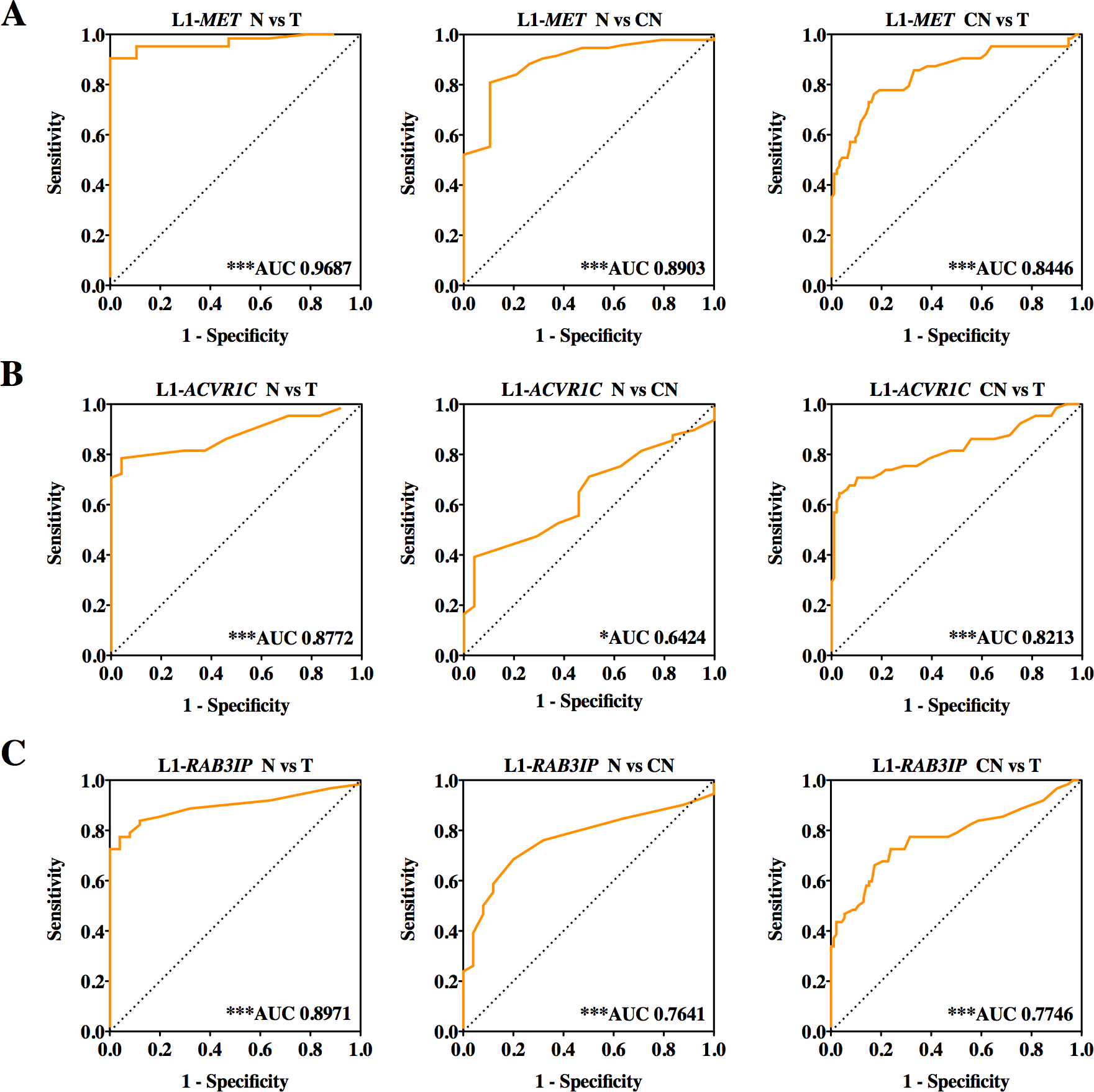

Supplement: Figure S8 — ROC curves for specific L1s. (A) ROC curves using L1-MET methylation distinguish between normal bladder tissue (N) and corresponding normal bladder tissues (CN), N and bladder tumors (T), and CN and T. (B) ROC curves using L1-ACVR1C methylation, and (C) ROC curves using L1-RAB3IP methylation. *** represents p<0.001 and * represents p<0.05. (0.65 MB TIF) [file pgen.1000917.s008.tif]

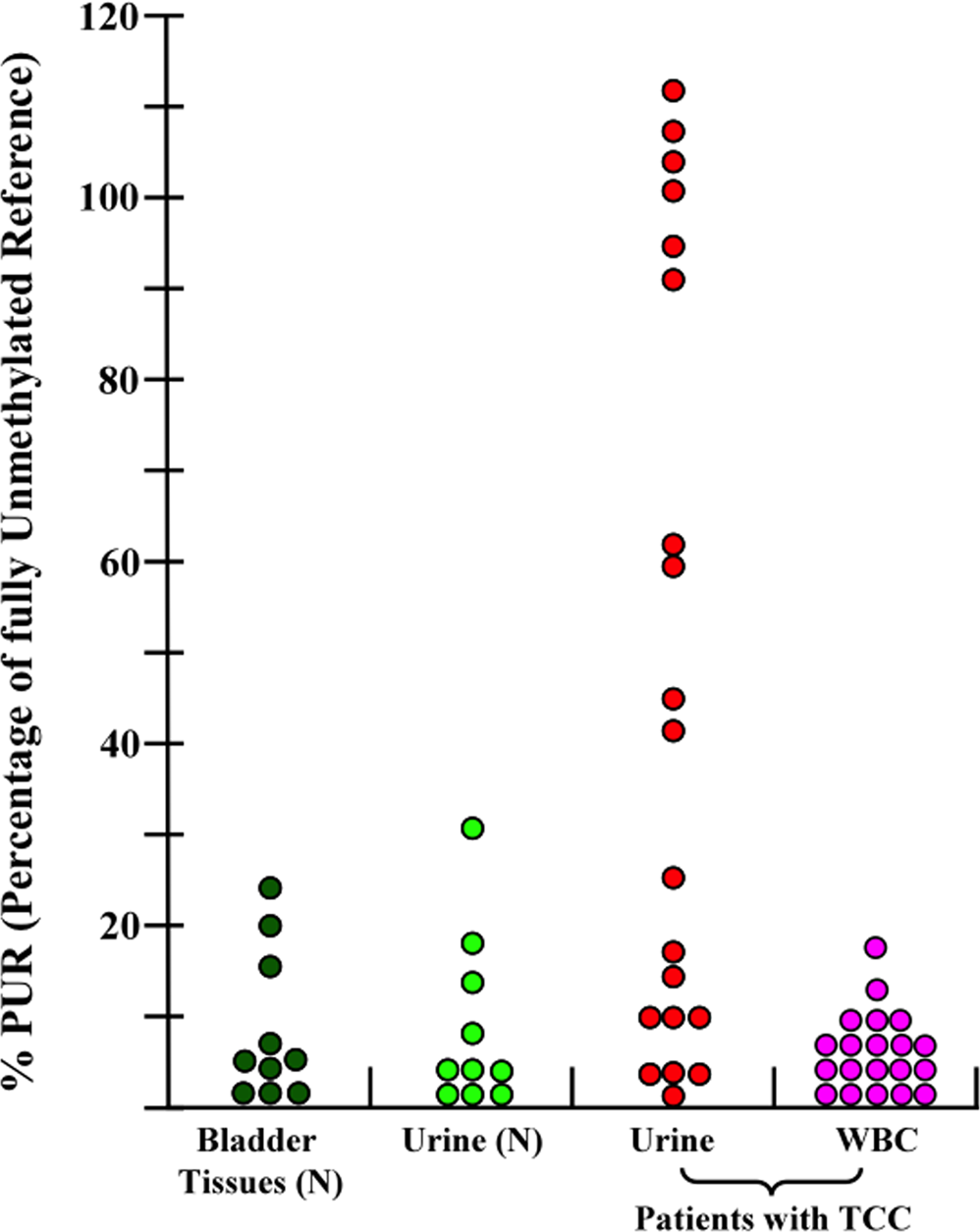

Supplement: Figure S9 — Detection of L1-MET hypomethylation in urine sediments of patients with bladder cancer. Bisulfite-specific primers and a probe were designed for the MethyLight assay that amplified only completely unmethylated strands of L1-MET. Bladder tissues (N) from age-matched patients without bladder cancer (n = 10) and urine (N) from age-matched healthy volunteers (n = 10) showed low levels of L1-MET hypomethylation. However, urine (n = 20) from patients with TCC showed high levels of L1-MET hypomethylation, which was specific to the bladder since it was not detected in their white blood cells (WBC) (n = 20). Unmethylated levels (Y axis) indicate the Percent of fully Unmethylated Reference (PUR) values. (0.30 MB TIF) [file pgen.1000917.s009.tif]

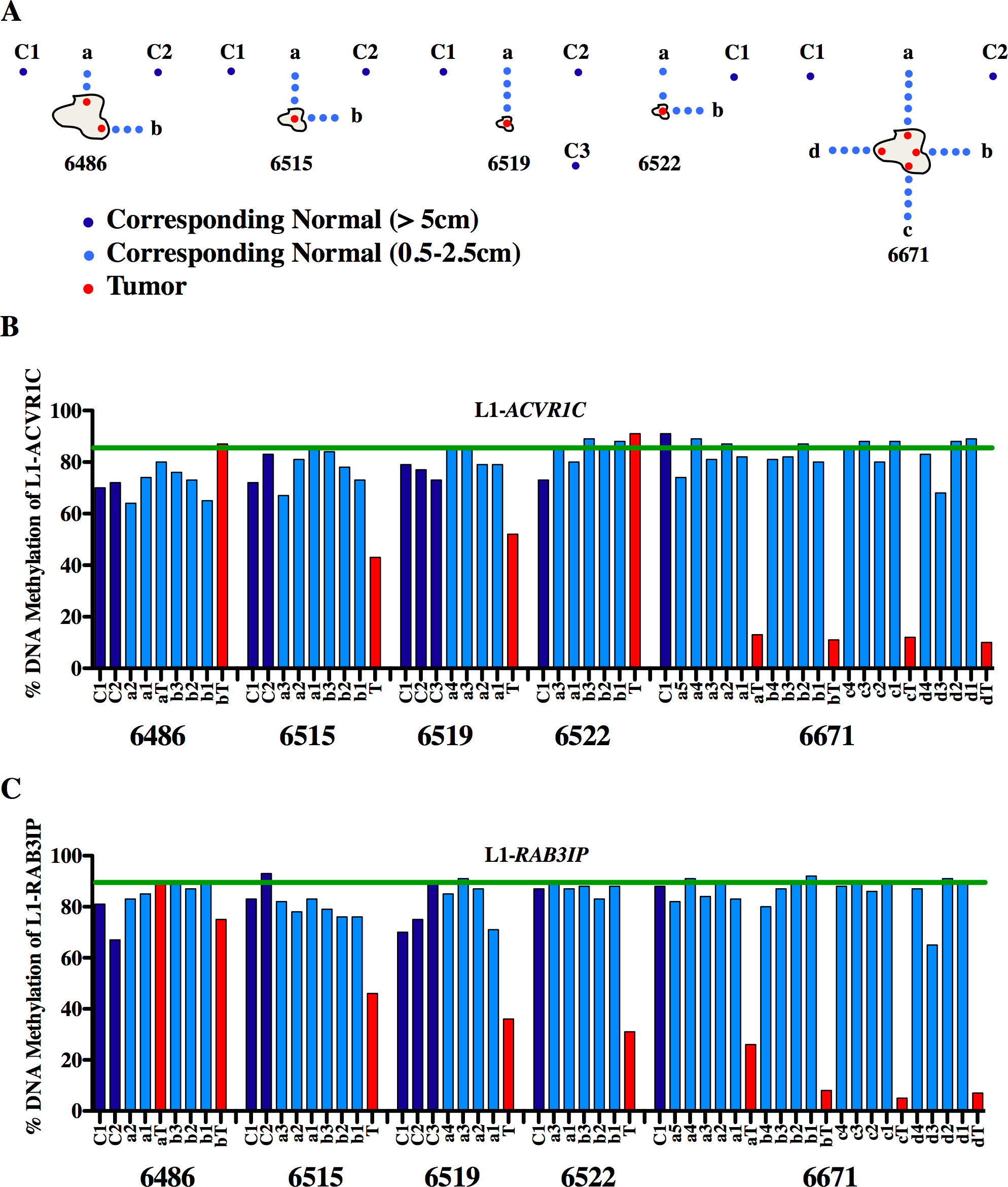

Supplement: Figure S10 — Methylation of specific L1s across the bladder. (A) Tissue samples were taken from five patients of their tumors (red, T) and at increasing distances from the tumor (0.5 to 2 cm) in the surrounding normal-appearing tissue in multiple directions (light blue, a to d). Additionally, distant normal-appearing samples were taken at least 5 cm from the tumor (dark blue, C). (B) Methylation at L1-ACVR1C and (C) L1-RAB3IP was measured by pyrosequencing. The green line represents the mean methylation of 12 normal samples from cancer-free patients. While there are no error bars for the clinical sample analysis due to the extremely limited amount of sample DNA. the results show a consistent trend. (1.62 MB TIF) [file pgen.1000917.s010.tif]

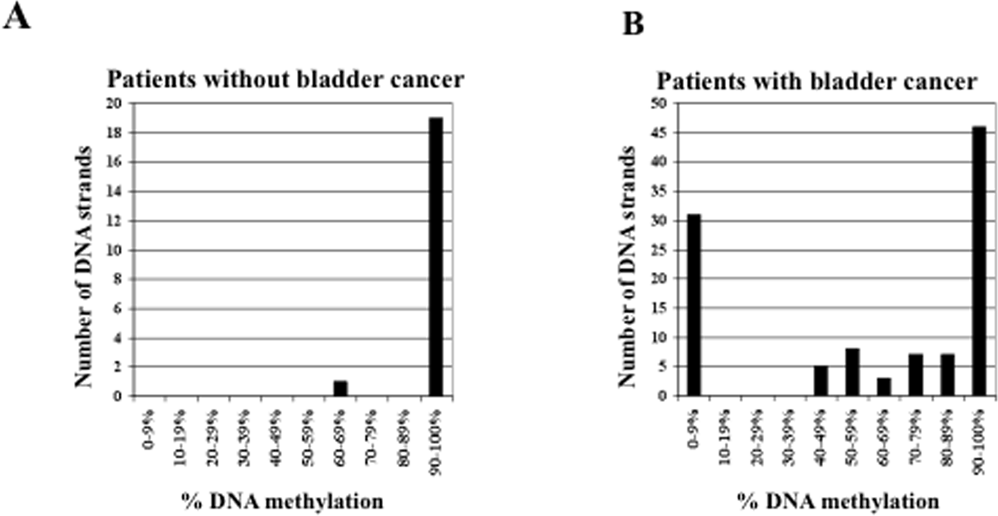

Supplement: Figure S11 — Bisulfite sequencing of L1-MET. Biphasic distribution of L1-MET methylation status in corresponding tissue from a patient with bladder cancer is revealed by plotting the number of DNA strands by the percent of CpG sites methylated. (0.18 MB TIF) [file pgen.1000917.s011.tif]

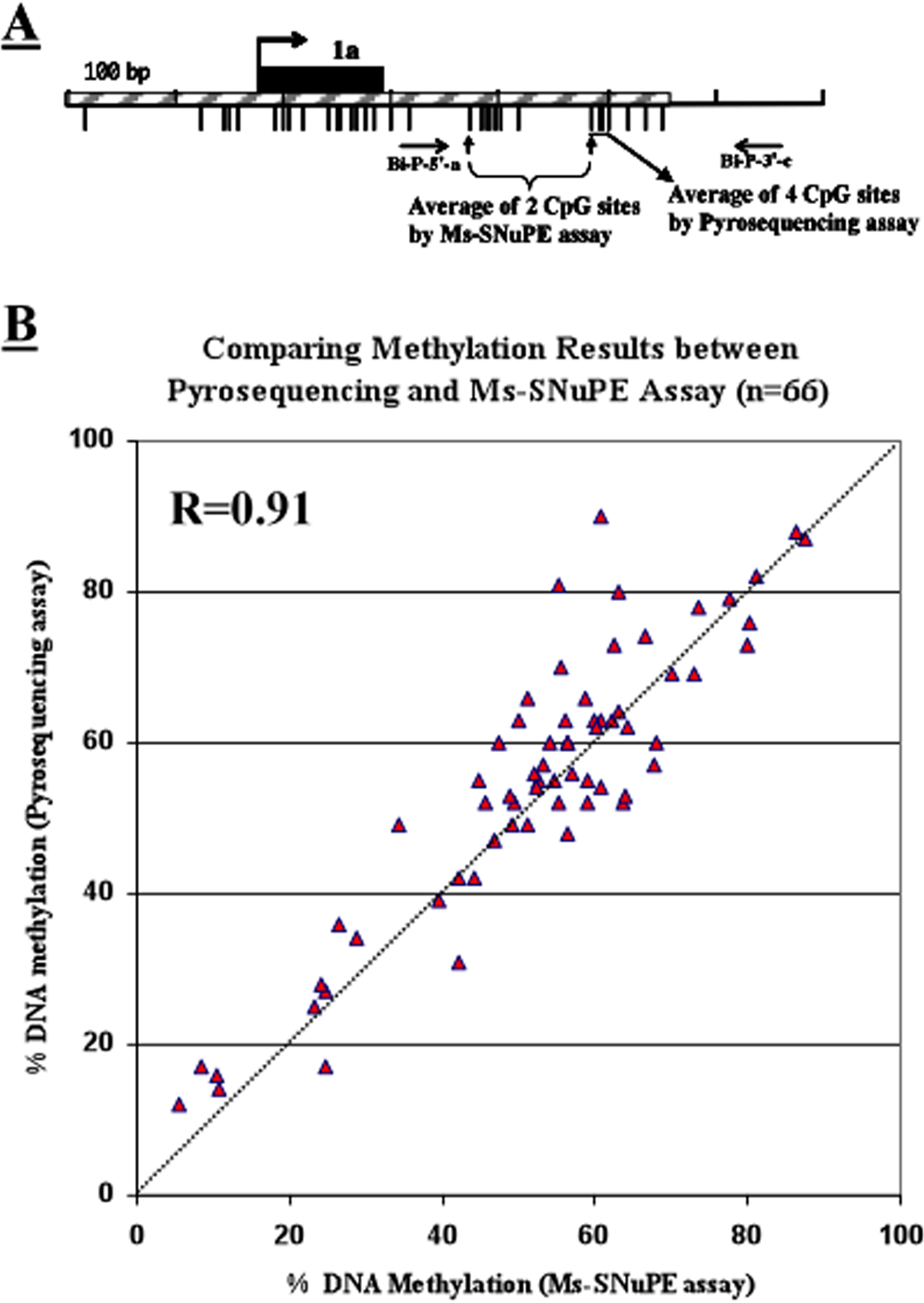

Supplement: Figure S12 — Ms-SNuPE and pyrosequencing yield similar methylation results. While both Ms-SNuPE and Pyrosequencing are quantitative assays, Pyrosequencing is much more high throughput. Therefore, we developed a Pyrosequencing assay for the rest studies. (A) We measured 4 CpG sites by Pyrosequencing assay in contrast with the 2 CpG sites by Ms-SNuPE. (B) We randomly chose 66 samples previously analyzed by Ms-SNuPE to perform Pyrosequencing on and the results are very similar from both assays (R = 0.91). (0.42 MB TIF) [file pgen.1000917.s012.tif]
