# Supplementary material for: Hypomethylation of a LINE-1 Promoter Activates an Alternate Transcript of the MET Oncogene in Bladders with Cancer
Source: PLoS Genet. 2010 Apr 22;6(4):e1000917. doi: 10.1371/journal.pgen.1000917 (PMC2858672; doi:10.1371/journal.pgen.1000917)
Supplement: Table S1 — Primer sequences. (0.08 MB DOC) [file pgen.1000917.s013.doc]

Supplemental Table 1. Primer sequences.

| **MS-SNuPE Primers** | | |
| --- | --- | --- |
|  | **L1-*MET*** | **Global L1** |
| Sense | 5'-GTGTTTTTTAAGTGAGGTAATGTT | 5'-GTGTTTTTTAAGTGAGGTAATGTT |
| Antisense | 5'-ATCCAACCACTACAAACTAC | 5'-CATCTAAAATACTAAATTCATCTCACTAA |
| Sequencing | S1: 5'-TTTTAAGTGA GGTAATGTTT | S1: 5'-TTTTAAGTGA GGTAATGTTT |
|  | S2: 5'-AGATGGAAATGTAGAAATTATT | S2: 5'-AGATGGAAATGTAGAAATTATT |
|  |  |  |
| **Pyrosequencing Primers** | | |
|  | **L1-*MET*** | **Global L1** |
| Sense | 5'-GTGTTTTTTAAGTGAGGTAATGTT | 5' TTTTGAGTTAGGTGTGGGATATA |
| Antisense | 5'-BIOTIN-ATCCAACCACTACAAACTAC | 5'-BIOTIN-AAAATCAAAAAATTCCCTTTC |
| Sequencing | 5'-AGATGGAAATGTAGAAATTATT | 5'-AGTTAGGTGTGGGATATAGT |
|  | **L1-*ACVR1C*** |  |
| Sense | 5'-GAAAGGGAATTTTTTGATTTTTTG |  |
| Antisense | 5-BIOTIN-AACACATAATACAAAACTATATTCACAA |  |
| Sequencing | 5'-TTTTTAGGTG AGGTAATGTTT |  |
|  | **L1-*RAR3IP*** |  |
| Sense | 5'-GAAAGGGAATTTTTTGATTTTTTG |  |
| Antisense | 5'-BIOTIN-ACTTACAAAATCTATAAAATAACATC |  |
| Sequencing | 5'-TTTAGATGGA AATGTAGAAATTATT |  |
|  |  |  |
| **Bisulfite Sequencing Primers** | | |
|  | **L1-*MET* (clinical samples)** | **LI-*MET* (MSPA)** |
| Sense | 5’-GTGTTTTTTAAGTGAGGTAATGTT | 5’-TGTTAGATAGGGATATTTAAGTT |
| Antisense | 5’-ATCCAACCACTACAAACTAC | 5’-CTCAAAAATCTAAATAAATTCTAAATTTT |
|  |  |  |
| **Southern Probe Primers** | | |
|  | **Global LI** |  |
| Sense | 5’-AGAAATCACCCGTCTTCTGC |  |
| Antisense | 5’-AATCATGGTCCTCCAACCAC |  |
|  |  |  |
| **ChIP Primers** | | |
|  | **L1-*MET*** | **Global L1** |
| Sense | 5'-CCGTCTTCTGCGTCGCTC | 5'-CCGTCTTCTGCGTCGCTC |
| Antisense | 5'-CAATCATGGTCCTCCAACCACTAC | 5'-CCAAGATGGCCGAATAGGAA |
| Probe | 5'-CACCCGTCTTCTGCGTCGCTCA | 5'-CACCCGTCTTCTGCGTCGCTCA |
|  | **L1-*ACVR1C*** |  |
| Sense | 5'-CCGTCTTCTGCGTCGCTC |  |
| Antisense | 5'-CTGTCCTAAAGTCTATGCTAGCTAGTGG |  |
| Probe | 5'-CACCCGTCTTCTGCGTCGCTCA |  |
|  | **L1-*RAR3IP*** |  |
| Sense | 5'-CCGTCTTCTGCGTCGCTC |  |
| Antisense | 5'-TGTTCGAGTAATTGGAGTTCAAGAGG |  |
| Probe | 5'-TCACGCTGGTAGCTGTAGACCGGAGCT |  |
|  |  |  |
| **Real-Time RT-PCR Primers** | | |
|  | ***MET*** | **L1-*MET*** |
| Sense | 5'-GATGTCTCCAGCATTTTTACGGAC | 5'-GGAGCCAGAGAGCCTAGGCTTAG |
| Antisense | 5'-CTGTGGTAAACTCTGTTCGATATTCATC | 5'-CTGTGGTAAACTCTGTTCGATATTCATC |
| Probe | 5'-ACTTCTGAGAAATTCATCAGGCTGTGAAGCG | 5'-ACTTCTGAGAAATTCATCAGGCTGTGAAGCG |
|  | ***ACVR1C*** | **L1-*ACVR1C*** |
| Sense | 5'-CAAAACCGAATGCTGCTTCAC | 5'-TCGGGTGGGAGTGACCC |
| Antisense | 5'-GCACAGTAATAATGATGGCCAGC | 5'-GCACAGTAATAATGATGGCCAGC |
| Probe | 5'-TGCCCCAAAACTTGGACCCATGG | 5'-TGCCCCAAAACTTGGACCCATGG |
|  | ***RAR3IP*** | **L1-*RAR3IP*** |
| Sense | 5'-GTTGAGTACTGATAGTCTGTCTCGTTTACG | 5'-TCCGTGGGCGTAGGACC |
| Antisense | 5'-GAGCTTCCTCAAATAGACTAGCTGTGAG | 5'-GAGCTTCCTCAAATAGACTAGCTGTGAG |
| Probe | 5'-TGCGAGATCAACTTGGACA (MGB) | 5'-TGCGAGATCAACTTGGACA (MGB) |
|  | ***GAPDH*** |  |
| Sense | 5'-TGAAGGTCGGAGTCAACGG |  |
| Antisense | 5'-AGAGTTAAAAGCAGCCCTGGTG |  |
| Probe | 5'-TTTGGTCGTATTGGGCGCCTGG |  |
|  |  |  |
| **RLM-RACE Primers** | | |
|  | L1-MET |  |
| Inner | 5'-CTTCACAGCCTGATGAATTTCTCAGAAGT |  |
| Outer | 5'-CTGTGGTAAACTCTGTTCGATATTCATCAC |  |
